# The *Clostridium botulinum* C2 Toxin Subunit C2IIa Delivers Enzymes with Positively Charged N-Termini into the Cytosol of Target Cells

**DOI:** 10.3390/toxins15060390

**Published:** 2023-06-09

**Authors:** Sebastian Heber, Joscha Borho, Nicole Stadler, Fanny Wondany, Irina König, Jens Michaelis, Panagiotis Papatheodorou, Holger Barth, Maximilian Fellermann

**Affiliations:** 1 Institute of Experimental and Clinical Pharmacology, Toxicology and Pharmacology of Natural Products, Ulm University Medical Center, 89081 Ulm, Germany; 2 Institute of Biophysics, Ulm University, 89081 Ulm, Germany

**Keywords:** bacterial AB-type protein toxins, cytosolic drug delivery; molecular Trojan horse, *Clostridium botulinum* C2 toxin, C2IIa, polyhistidine-tag; protective antigen (PA)

## Abstract

The binary *Clostridium* (*C.*) *botulinum* C2 toxin consists of two non-linked proteins. The proteolytically activated binding/transport subunit C2IIa forms barrel-shaped homoheptamers, which bind to cell surface receptors, mediate endocytosis, and translocate the enzyme subunit C2I into the cytosol of target cells. Here, we investigate whether C2IIa can be harnessed as a transporter for proteins/enzymes fused to polycationic tags, as earlier demonstrated for the related anthrax toxin transport subunit PA_63_. To test C2IIa-mediated transport in cultured cells, reporter enzymes are generated by fusing different polycationic tags to the N- or C-terminus of other bacterial toxins’ catalytic A subunits. C2IIa as well as PA_63_ deliver N-terminally polyhistidine-tagged proteins more efficiently compared to C-terminally tagged ones. However, in contrast to PA_63_, C2IIa does not efficiently deliver polylysine-tagged proteins into the cytosol of target cells. Moreover, untagged enzymes with a native cationic N-terminus are efficiently transported by both C2IIa and PA_63_. In conclusion, the C2IIa-transporter serves as a transport system for enzymes that harbor positively charged amino acids at their N-terminus. The charge distribution at the N-terminus of cargo proteins and their ability to unfold in the endosome and subsequently refold in the cytosol determine transport feasibility and efficiency.

## 1. Introduction

The C2 toxin from *Clostridium* (*C.*) *botulinum* is a binary AB-type protein toxin that ADP-ribosylates actin in the cytosol of target cells [1,2]. It is composed of two non-linked subunits called C2I and C2II, which have to be combined for the full cytotoxic potential to evolve [3]. C2II is the binding/translocation subunit (B-subunit) with a molecular weight of about 80 or 100 kDa, depending on the producing *C. botulinum* strain [4,5]. It must be proteolytically cleaved to obtain biologically active C2IIa (~60 or 80 kDa), which can be simulated in vitro by trypsin treatment [3,4,5]. C2IIa binds to asparagine-linked carbohydrate receptors [6] present in all mammalian cell types and forms homoheptameric barrel-shaped complexes [5]. These C2IIa oligomers assemble with the enzymatically active subunit (A-subunit) C2I (~50 kDa), and the C2IIa/C2I toxin complexes are internalized from the cell surface into endosomal vesicles by receptor-mediated endocytosis [7,8]. Following acidification of the endosomes, C2IIa changes its conformation and inserts as a pore into the endosomal membrane, which serves as a transmembrane channel for the translocation of C2I from the endosomal lumen into the cytosol [2,5]. In the cytosol, C2I ADP-ribosylates globular actin at arginine-177 [9], resulting in depolymerization of the actin cytoskeleton [5,10], cell rounding [11], and delayed caspase-dependent apoptotic cell death [12].

The structure and mode of action of C2II is highly similar to the anthrax toxin’s B-subunit called protective antigen (PA_83_), produced and secreted by *Bacillus anthracis* [2,13]. PA_83_ has a molecular weight of 83 kDa and must be proteolytically activated to obtain biologically active PA_63_ (63 kDa), which forms heptameric or octameric barrel-shaped oligomers [14,15]. Both C2II and PA_83_ can be subdivided into four functionally different domains (1–4) [15,16]. The N-terminal domain 1 of C2II and PA_83_ is important for correct protein folding of the complete B-subunits, contains the proteolytic activation site, and most probably serves as a docking site for the respective A-subunits on top of the oligomer after proteolytic activation [15,16,17,18]. Domain 2 is important for membrane insertion, pore formation, and translocation of the A-subunits into the cytosol [5,15,18,19,20]. Domain 3 is most probably involved in the oligomerization of C2IIa and PA_63_ [18,21], and domain 4 mediates the binding to the respective cell surface receptor [16,22,23]. Since PA_63_ binds to other cell surface receptors (anthrax toxin receptor/tumor endothelial marker 8 and capillary morphogenesis protein 2 [24,25,26]) compared to C2IIa, the domains 4 of PA_63_ and C2IIa share no homology [2,16]. In contrast, the domains 1–3 of PA_83_ and C2II possess a sequence homology of about 50% [2,16].

Due to their binary composition and highly useful ability to deliver enzymes into the cytosol of a target cell, PA_63_ and C2IIa have been harnessed as protein delivery tools for various foreign cargo molecules [18,27,28]. Therefore, the catalytically inactive and thus non-toxic N-termini of the A-subunits (C2I_N_ for C2I and LF_N_ for the anthrax toxin A-subunit lethal factor (LF)) are exploited as adapters, which bind to their respective B-subunits and mediate the transport of the fused cargo proteins into the cytosol of target cells [27,29]. In case of the C2 toxin, this strategy has been used, among others, for cytosolic delivery of a Rho GTPase inhibitor (C3 toxin from *C. botulinum*) [29], the p53 tumor suppressor [28], or different biotinylated cargo molecules [30]. Using LF_N_ as an adapter for PA_63_-mediated delivery represents a more established strategy, comprising a large variety of cargo molecules (for review, see [27]) including the A-subunit of the diphtheria toxin (DTA) [31], dihydrofolate reductase [32], a Ras/Rap1-specific endopeptidase [33], and Cas9 endonuclease [34]. Moreover, for the PA_63_ transport system, it has been shown that cargo proteins with an N-terminal polycationic tag are also efficiently delivered into the cytosol of target cells [35]. By using DTA as cargo molecule, it is found that transport efficiency increases with tag length and that hexalysine-tagged proteins are about 100-fold more efficiently delivered compared to hexahistidine-tagged ones [35]. Nevertheless, due to its broad application in the purification of recombinantly expressed proteins, the polyhistidine-tag is more commonly used for PA_63_-mediated delivery. The actin ADP-ribosylating enzyme domain of the *Photorhabdus luminescens* toxin complex (TccC3hvr) [36], the tumor metastasis suppressor protein nucleoside diphosphate kinase A [37], and also the Cas9 endonuclease [34] are cargo molecules, which are polyhistidine-tagged and delivered via PA_63_ into the cytosol of cells. However, the delivery of proteins with polycationic tags was, to date, only shown for PA_63_ and not for C2IIa. In contrast, by indirectly comparing the binding of untagged or His-tagged cargo molecules to PA_63_ or C2IIa, clear differences were observed for the B-subunits in black lipid bilayer experiments in vitro [38]. All five His-tagged proteins tested by Beitzinger et al. showed increased binding to PA_63_ compared to their untagged versions. In contrast, only for one of the five tested cargo molecules did the binding affinity for C2IIa increase remarkably in the presence of an N-terminal His-tag [38]. However, these results for C2IIa were not confirmed in cell-based assays or via direct protein–protein interaction studies.

In the present study, the C2IIa-mediated protein delivery of polycationic-tagged proteins is investigated in more detail and compared to the transport of cargo proteins by PA_63_. For a better comparison of C2IIa and PA_63_, DTA is used as an initial cargo model, and positively charged tags are attached to either its N- or C-terminus. C2IIa delivers N-terminally His-tagged DTA more efficiently into the cytosol of HeLa cells compared to C-terminally His-tagged DTA. These results are confirmed by using the glucosyltransferase domain of toxin B (TcdB-GTD) from *Clostridioides difficile* as a second cargo model. Interestingly, a fundamental difference between C2IIa and PA_63_ is found in the delivery of N-terminally polylysine-tagged cargo proteins. Moreover, investigating the transport of an eGFP-labeled His_DTA reveals that this stably folded cargo molecule does not reach the cytosol of target cells despite efficient endosomal uptake, most probably because the eGFP part is unable to unfold within endosomes, which is necessary for PA_63_- and C2IIa-mediated translocation into the cytosol. Finally, completely untagged TccC3hvr with a natively positively charged N-terminus is efficiently delivered into the cytosol of HeLa cells via both transporters, PA_63_ and C2IIa. In conclusion, the translocation of cargo proteins occurs through the C2IIa/PA_63_ pores, requires the ability of the cargo protein to unfold/refold, and depends on the presence as well as the position of cationic amino acids.

## 2. Results

To investigate whether C2IIa can deliver N-terminally His-tagged proteins into the cytosol of target cells, we used various cargo molecules and compared their delivery with that of the established PA_63_ transport machinery.

### 2.1. N-Terminal Polyhistidine-Tagging of DTA Mediates Cytosolic Delivery via C2IIa

**Figure 1:**
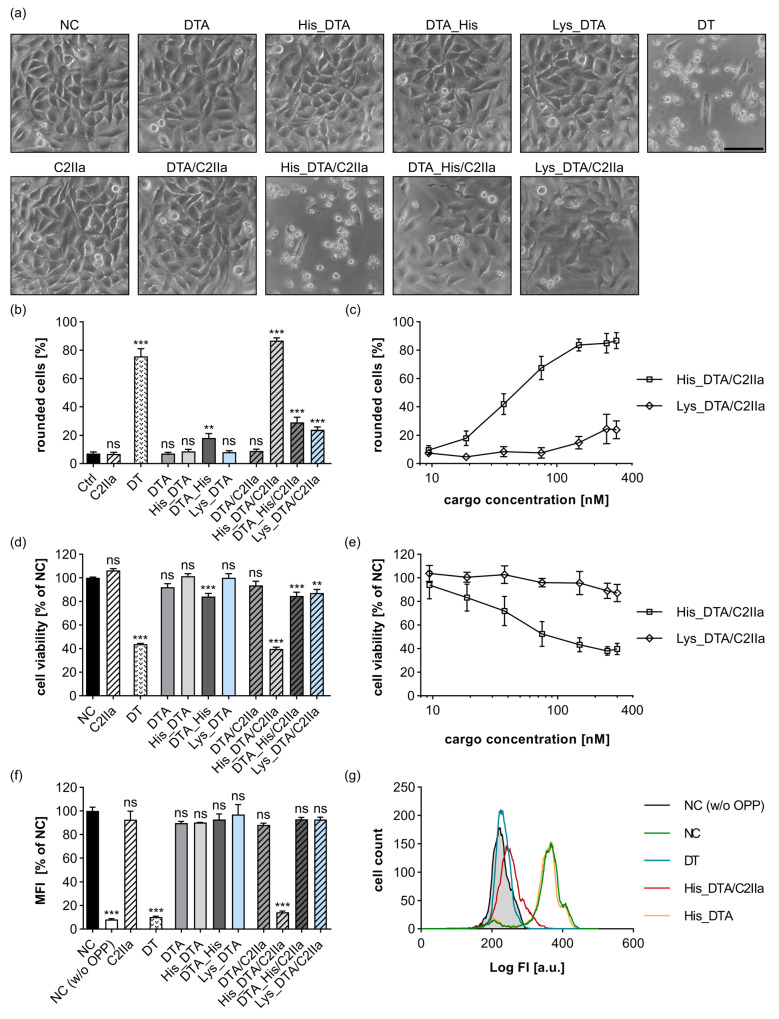
His_DTA is transported into HeLa cells by C2IIa in the presence of an N-terminal His-tag. (**a**) HeLa cells were treated as indicated with 25 nM C2IIa, 300 nM DTA variants, 0.25 nM DT, or were left untreated (NC). (**a**) Representative pictures after 24 h incubation time with indicated proteins are depicted. Scale bar corresponds to 100 µm. (**b**) HeLa cells were treated as indicated with identical concentrations as in (**a**). Pictures were taken after 24 h, and the percentage of rounded cells was quantified of total cell number. Values are given as mean ± SD (*n* = 9) of triplicates from three individual experiments. (**c**) Depicted is the percentage of rounded cells after 24 h in the same experiments as in (**b**) for a constant concentration of 25 nM C2IIa in combination with increasing His_DTA and Lys_DTA concentrations. His_DTA and Lys_DTA concentrations were 9.38 nM, 18.75 nM, 37.50 nM, 75 nM, 150 nM, 250 nM, and 300 nM. Values are given as mean ± SD (*n* = 9) of triplicates from three individual experiments. (**d**) Relative viability (% of NC) of the cells from (**b**) measured via MTS assay. Values are given as mean ± SD (*n* = 9) of triplicates from three individual experiments. (**e**) Relative viability (% of NC) of the cells from (**c**) measured via MTS assay. Values are given as mean ± SD (*n* = 9) of triplicates from three individual experiments. (**f**) HeLa cells were treated as indicated with 25 nM C2IIa, 125 nM DTA cargo, 2.5 nM DT, or left untreated (NC). To analyze protein synthesis 4 h after treatment, O-propargyl-puromycin (OPP) was added to all samples except for an untreated control (NC (w/o OPP)) which represents background fluorescence. The median fluorescence intensity (MFI) values of all samples were measured via flow cytometry. Values are given as mean ± SD (*n* = 2) of duplicates from one representative experiment out of three replicates. (**g**) Representative histogram with fluorescence intensity (FI) values of the flow cytometric measurement depicted in (**f**). (**b**,**d**,**f**) Statistical analysis was performed compared to the NC by using non-parametric one-way ANOVA in combination with Dunnett’s correction for multiple comparison as described under Section 5.12 in Section 5 (ns *p* ≥ 0.05, ** *p* < 0.01, *** *p* < 0.001).

Analogous to the literature about PA_63_-mediated transport [35], DTA (amino acids 1-193 without signal sequence) was used as a first cargo molecule. DTA is an ideal reporter enzyme for cytosolic delivery, since successful cytosolic release will result in specific ADP-ribosylation of the eukaryotic elongation factor 2 (eEF2), causing inhibition of protein synthesis [39,40,41,42], and lead to a characteristic change in HeLa cell morphology, i.e., cell rounding [43,44,45]. Both effects, cell rounding and inhibition of protein synthesis, are easy to analyze. To investigate the cytosolic delivery of this reporter enzyme, we generated DTA variants without any tag (DTA), with N- or C-terminal 6x His-tag (His_DTA, DTA_His), or with N-terminal 6x Lys-tag (Lys_DTA) (for primary structures, see Supplementary Figure S1a). After sequence validation, the proteins were expressed and purified, and the purity and identity were analyzed (Supplementary Figure S2); it was confirmed that all DTA variants are catalytically active and can therefore be used as reporter enzymes (Supplementary Figure S3). By treating HeLa cells with these DTA cargo proteins or C2IIa alone, only neglectable changes in cell morphology (Figure 1a,b) or cell viability (Figure 1d) were detected. In contrast, treatment of HeLa cells with the combination of C2IIa and His_DTA resulted in cell rounding (Figure 1a,b) and the drastic reduction of cell viability (Figure 1d), which was comparable to samples treated with wild type diphtheria toxin (DT) as positive control. However, treatment of HeLa cells with the combination of C2IIa together with DTA, DTA_His, or Lys_DTA, respectively, did not result in such clear changes in cell morphology or viability as those seen for C2IIa together with His_DTA (Figure 1a,b,d). Moreover, these results were confirmed by analyzing the intracellular protein synthesis via flow cytometry, where only for the combination His_DTA/C2IIa or wild type DT could a strong reduction in protein synthesis be detected (Figure 1f,g). These results indicate that C2IIa can be used to deliver His-tagged cargo proteins into the cytosol of target cells and that the position of the His-tag at the N-terminus is highly important for C2IIa-mediated delivery.

Moreover, in the cell rounding and cell viability assays, it was shown that cytosolic delivery of His_DTA via C2IIa is strongly dependent on the His_DTA concentration (Figure 1c,e) as well as on the C2IIa concentration (Supplementary Figure S6).

Blanke et al. showed that Lys_DTA is approximately 100-fold more effectively delivered via PA_63_ compared to His_DTA [35]. We reproduced their experiments with our DTA constructs and obtained consistent data for the transport of His_DTA and Lys_DTA via PA_63_ (Figure S7). Notably, the complete opposite was observed for C2IIa-mediated delivery of Lys_DTA in direct comparison to His_DTA (Figure 1c,e). Only at Lys_DTA concentrations of above 150 nM were moderate effects on cell morphology or cell viability visible for the combination Lys_DTA/C2IIa, while for His_DTA/C2IIa, the first effects were visible from 18.75 nM His_DTA. Despite this difference in PA_63_- and C2IIa-mediated transport, we found that both B-subunits were able to deliver His-tagged proteins into the cytosol of target cells.

### 2.2. Delivery of His_DTA Is Dependent on C2IIa-Mediated Internalization, Endosomal Acidification, and Translocation through the C2IIa Pore

Prompted by this result, the underlying mechanism of the C2IIa-mediated transport of His_tagged proteins into cells was investigated in more detail. An important prerequisite of C2IIa-mediated delivery is the interaction of the C2IIa pore with the cargo molecule. Direct interaction of C2IIa with His_DTA, DTA_His, and Lys_DTA, but not with DTA, was observed in a dot blot experiment by spotting C2IIa and performing an overlay with the DTA variants (Supplementary Figure S8). The specificity of this binding was validated by spotting BSA, which did not bind to the proteins in the overlay. Hence, we conclude that a positively charged tag (polyhistidine or polylysine) is needed for the interaction with C2IIa, and this interaction occurs independently of the position of the tag.

**Figure 2:**
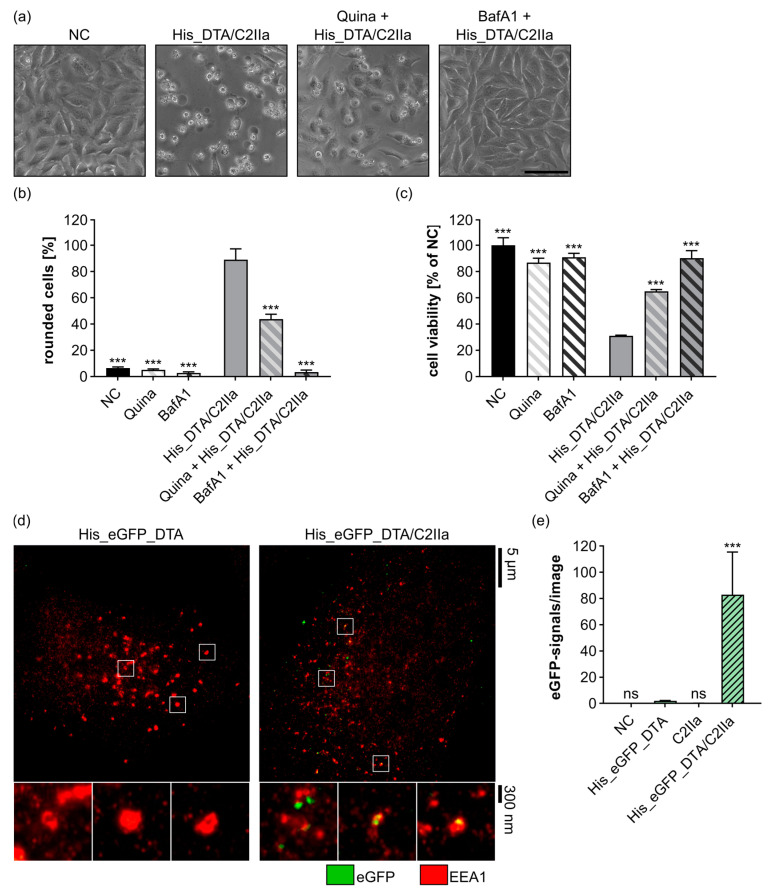
Endocytosis and cytosolic release of His-tagged cargo proteins depends on the uptake and translocation via C2IIa. (**a**) HeLa cells were treated as indicated with 25 nM C2IIa, 250 nM His_DTA, 5 µM Quina, 100 nM BafA1, or left untreated (NC). Representative pictures after 24 h incubation time are depicted. Scale bar corresponds to 100 µm. (**b**) HeLa cells were treated as indicated with the same concentrations as in (**a**). Pictures were taken after 24 h, and the percentage of rounded cells was quantified. Values are given as mean ± SD (*n* = 3) of triplicates from one representative experiment out of three replicates. (**c**) Relative viability (% of NC) of the cells from (**b**) measured via MTS assay. Values are given as mean ± SD (*n* = 3) of triplicates from one representative experiment out of three replicates. (**b**,**c**) Statistical analysis was performed compared to the treatment with His_DTA/C2IIa by using non-parametric one-way ANOVA in combination with Dunnett’s correction for multiple comparison as described under Section 5.12 in Section 5 (*** *p* < 0.001). (**d**) HeLa cells were treated for 30 min with 250 nM His_eGFP_DTA or a combination of 25 nM C2IIa and 250 nM His_eGFP_DTA. STED super-resolution microscopy was performed, and representative micrographs are depicted together with three magnified areas below the image (white squares indicate the respective regions in the main image). eGFP-signals are shown in green, while EEA1-signals are depicted in red, thus overlapping signals appear in yellow. Scale bars correspond to 5 µm for the main images or 300 nm for the magnified areas. (**e**) Cells were treated as in (**d**), with 25 nM C2IIa, or left untreated (NC). The eGFP-signals, recorded via STED microscopy, were quantified (Material and Methods Section 5.10) and depicted in a column diagram. Values are given as mean ± SD (*n* = 5) from five individual experiments. Statistical analysis was performed compared to the treatment with His_eGFP_DTA by using non-parametric one-way ANOVA in combination with Dunnett’s correction for multiple comparison as described under Section 5.12 in Section 5 (ns *p* ≥ 0.05, *** *p* < 0.001).

In the next step, the cellular uptake of His-tagged cargo and C2IIa was investigated in more detail. Hence, we treated HeLa cells with the established C2IIa pore-blocker quinacrine (Quina) [46,47] and the combination His_DTA/C2IIa (Figure 2a–c). Quina treatment blocked the cytosolic release of His_DTA as indicated by hampered cell rounding (Figure 2a,b) and diminished loss of cell viability (Figure 2c) compared to cells treated only with the combination His_DTA/C2IIa. Moreover, even stronger inhibition of His_DTA/C2IIa-mediated toxicity was detected when using Bafilomycin A1 (BafA1), a well-established inhibitor of endosomal acidification [5,48] (Figure 2a–c). These data indicate that the cytosolic delivery of His-tagged protein cargo via C2IIa is dependent on endosomal acidification and translocation through the C2IIa pore. The uptake into early endosomes was further investigated via STED super-resolution microscopy (Figure 2d,e) and by using His-tagged and eGFP-labeled DTA (His_eGFP_DTA). Already after 30 min of incubation, His_eGFP_DTA was internalized when HeLa cells were treated with the combination His_eGFP_DTA/C2IIa (Figure 2d).

As shown in the magnification of the STED images, the green eGFP-signals of the His_eGFP_DTA/C2IIa treatment were mainly found in close proximity to red-stained early endosomal antigen 1 (EEA1), indicating internalization of the toxin complex into early endosomes (Figure 2d). In contrast, cells treated with His_eGFP_DTA alone showed only neglectable internalization of the cargo molecule. The dependency on C2IIa-mediated uptake became even clearer by quantifying the eGFP-signals from the STED super-resolution microscopic pictures (Figure 2e and Supplementary Figure S9). No eGFP-signals were detected in the controls for C2IIa treatment alone or untreated cells (NC). In conclusion, C2IIa mediates the internalization of His_eGFP_DTA into early endosomes.

### 2.3. Stably Folded Cargo Proteins Such as eGFP Are Not Translocated into the Cytosol

**Figure 3:**
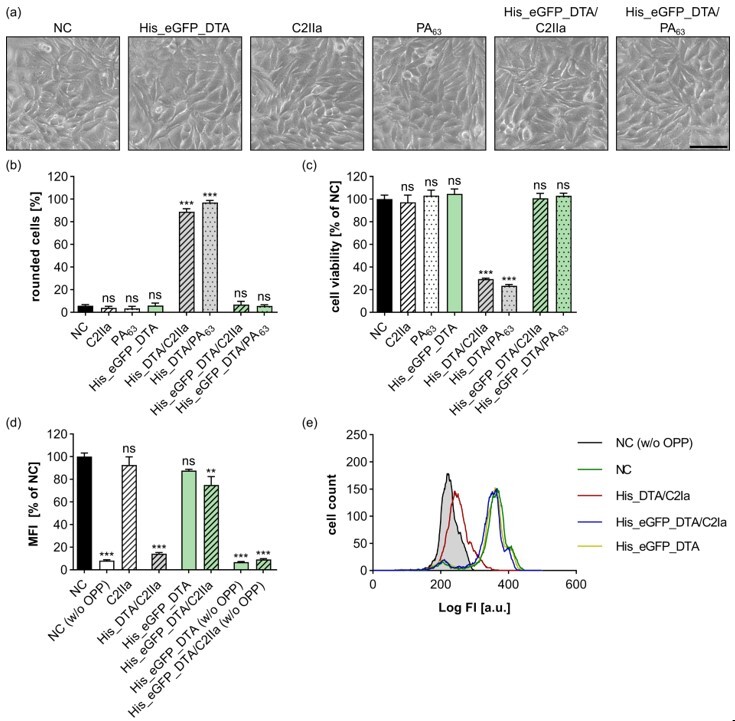
His_eGFP_DTA is not efficiently delivered into the cytosol of HeLa cells by C2IIa. (**a**) HeLa cells were treated as indicated with 25 nM C2IIa, 25 nM PA_63_, 250 nM His_eGFP_DTA, or left untreated (NC). Representative pictures after 24 h incubation time are depicted. Scale bar corresponds to 100 µm. (**b**) HeLa cells were treated as indicated with identical concentrations as in (**a**) and 250 nM His_DTA. Pictures were taken after 24 h, and the percentage of rounded cells was quantified. Values are given as mean ± SD (*n* = 3) of triplicates from one representative experiment of three replicates. (**c**) Relative viability (% of NC) of the cells from (**b**) measured via MTS assay. Values are given as mean ± SD (*n* = 3) of triplicates from one representative experiment of three replicates. (**d**) HeLa cells were treated as indicated with 25 nM C2IIa and 125 nM DTA cargo or left untreated (NC). To analyze protein synthesis 4 h after treatment, OPP was added to all samples except for the ones designated with (w/o OPP), which represent the background fluorescence, respectively. The median fluorescence intensity (MFI) values of all samples were measured via flow cytometry. Values are given as mean ± SD of (*n* = 2) duplicates from one representative experiment out of three replicates. The experiment was performed in parallel to the experiment in Figure 1f with the same controls. (**e**) Representative histogram with fluorescence intensity (FI) values of the flow cytometric measurement depicted in (**d**). (**b**–**d**) Statistical analysis was performed compared to the NC by using non-parametric one-way ANOVA in combination with Dunnett’s correction for multiple comparison as described under Section 5.12 in Section 5 (ns *p* ≥ 0.05, ** *p* < 0.01, *** *p* < 0.001).

For the investigation of endocytosis via fluorescence microscopy, His_eGFP_DTA is an ideal cargo protein. However, it was also shown that fusion of eGFP to cargo molecules can significantly reduce the transport efficiency via the PA_63_ pore [49]. To investigate the translocation of eGFP, the generated fusion protein His_eGFP_DTA is an optimal candidate. The DTA part provides simple and robust readouts for cytosolic release, while detection of single fluorescently labeled proteins in the cytosol would be hardly achievable. Even though the DTA part of this fusion protein tested enzymatically active (Supplementary Figure S3), neither for HeLa cells treated with the combination His_eGFP_DTA/C2IIa nor with the combination His_eGFP_DTA/PA_63_ were any changes in cell morphology (Figure 3a,b) or cell viability (Figure 3c) detected. Hence, fusion of eGFP to His_DTA blocked the translocation via C2IIa and PA_63_ completely, even when high His_eGFP_DTA concentrations (250 nM) were used. These results were also confirmed by analyzing the intracellular protein synthesis (Figure 3d,e). Treatment of HeLa cells with the combination of His_eGFP_DTA and C2IIa had only a neglectable effect on protein synthesis compared to His_DTA/C2IIa, which also held true for the single components C2IIa or His_eGFP_DTA alone. Taken together, the attachment of eGFP strongly inhibited the translocation of His_eGFP_DTA via the C2IIa pore.

### 2.4. Transport Evaluation with the Glucosyltransferase Domain of Toxin B

**Figure 4:**
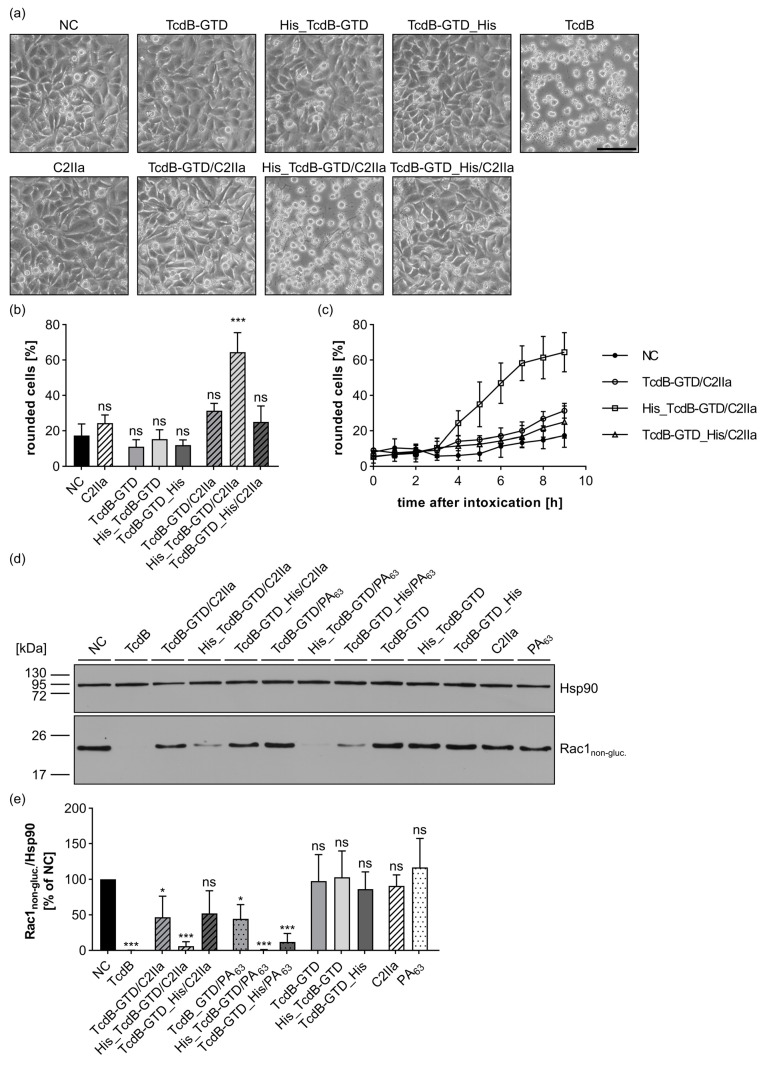
TcdB-GTD is efficiently transported into HeLa cells by C2IIa in the presence of an N-terminal His-tag. (**a**) HeLa cells were treated as indicated with 25 nM C2IIa, 100 nM TcdB-GTD cargo, 100 pM TcdB, or without toxin (NC). Representative pictures after 10 h incubation time are depicted. Scale bar corresponds to 100 µm. (**b**) HeLa cells were treated as indicated with identical concentrations as in (**a**). Depicted is the potion of rounded cells 9 h after intoxication. Values are given as mean ± SD (*n* = 3) of a triplicate from one representative experiment out of three replicates. (**c**) Quantification of rounded cells from the same experiment as in (**b**) at different time points. Values are given as mean ± SD (*n* = 3) of a triplicate from one representative experiment out of three replicates. (**d**) Probing of intracellular Rac1 glucosylation status in HeLa cell lysates. Cells were intoxicated as indicated. Proteins were used in identical concentrations as in (**a**). PA_63_ was used in a concentration of 10 nM. Cells were harvested after 10 h and transferred to SDS-PAGE and western blotting. Non-glucosylated Rac1 (Rac1_non-gluc._) was detected by immunoblotting, and Hsp90 served as loading control. Notably, a weak signal for Rac1_non-gluc._ indicated strong toxin activity in intact cells. (**e**) Quantification of the western blot from (**d**). The signal of Rac1_non-gluc._ was normalized to Hsp90 loading control and is given as percentage of NC. Values are given as mean ± SD (*n* = 4) of four biological replicates. (**b**,**e**) Statistical analysis was performed compared to NC by using non-parametric one-way ANOVA in combination with Dunnett’s correction for multiple comparison as described under Section 5.12 in Section 5 (ns *p* ≥ 0.05, * *p* < 0.05, *** *p* < 0.001).

Up to now, His_DTA has been the only His-tagged cargo protein delivered via C2IIa into the cytosol of target cells; no other tested DTA variant was efficiently transported. To exclude DTA-specific artifacts, we also investigated other cargo molecules. The glucosyltransferase domain of Toxin B (amino acids 1-543) was tested as a second cargo molecule. Similarly to DTA, an untagged (TcdB-GTD), an N-terminally His-tagged (His_TcdB-GTD), and a C-terminally His-tagged (TcdB-GTD_His) version were generated (for primary structures, see Supplementary Figure S1b). The purity and identity of all generated TcdB-GTD variants were confirmed (Supplementary Figure S2). Moreover, all constructs were proven to be enzymatically active and could therefore be used in cell-based in vitro assays (Supplementary Figure S4). When TcdB-GTD reaches the cytosol of target cells, it catalyzes the covalent transfer of glucose to small GTPases of the Ras/Rho-superfamily (glucosylation reaction) [50]. This modification of important Ras/Rho family members, e.g., RhoA, Rac1, or Cdc42, blocks their signal transduction, resulting in the disruption of the actin cytoskeleton and cell rounding [50,51]. Thereby, TcdB-GTD provides an easy morphological readout for cytosolic delivery as shown for the wild type Toxin B (TcdB) (Figure 4a). Furthermore, we chose this protein as the second reporter enzyme, since it is unrelated to the ADP-ribosyltransferases DTA or C2I (11% sequence identity with DTA and 16% with C2I, calculated via BLOSUM62 [52]). Treatment of HeLa cells with the TcdB-GTD variants alone did not result in any morphological change (for images, see Figure 4a; for quantification, see Figure 4b), indicating that these cargo molecules did not reach the cytosol by themselves. In contrast, treatment with C2IIa in combination with His_TcdB-GTD resulted in cell rounding of HeLa cells comparable to treatment with full-length toxin TcdB (Figure 4a,b). Moreover, rounding of cells treated with His_TcdB-GTD/C2IIa increased in a time-dependent manner from 3–9 h of incubation (Figure 4c). The cargo molecules TcdB-GTD and TcdB-GTD_His, respectively, only had a minor influence on cell rounding in combination with C2IIa (Figure 4a–c), highlighting the importance of the His-tag and its positioning at the N-terminus. Comparable results were obtained by using PA_63_ as the transporter instead of C2IIa (Supplementary Figure S10). Even though PA_63_ delivered TcdB-GTD_His more efficiently into the cytosol compared to untagged TcdB-GTD, this effect was much less efficient compared to the transport of His_TcdB-GTD (Supplementary Figure S10c). These results emphasize the similarity of both pores and that the position of the His-tag is also important for PA_63_-mediated delivery. All results for the different TcdB-GTD variants in combination with C2IIa or PA_63_, respectively, were validated on the molecular level by investigating the Rac1-glucosylation status via immunoblotting (Figure 4d,e). A primary antibody was used that specifically recognizes non-glycosylated Rac1 (Rac1_non-gluc._) but not glycosylated Rac1 (Figure 4d). Hence, a weak signal indicated strong TcdB-GTD intoxication of intact cells. In accordance with the earlier experiments, for the treatments with TcdB, as well as for the combinations His_TcdB-GTD/C2IIa, His_TcdB-GTD/PA_63_, and TcdB-GTD_His/PA_63_, a signal reduction and therefore successful cytosolic delivery was detected (for quantification, see Figure 4e). The stronger signal reduction for His_TcdB-GTD/PA_63_ compared to TcdB-GTD_His/PA_63_ confirms the cell morphology-based results that PA_63_ also delivers N-terminally His-tagged proteins more efficiently compared to C-terminally His-tagged ones. In conclusion, the presence and position of the His-tag is important for the delivery of the second reporter enzyme TcdB-GTD via C2IIa or PA_63_, respectively. Moreover, similar to the first tested cargo molecule DTA, the cytosolic release of His_TcdB-GTD is dependent on endosomal acidification and translocation through the C2IIa pore as shown with the established inhibitors BafA1 and Quina (Supplementary Figure S11).

### 2.5. Transport of Untagged Proteins with Natively Positively Charged N-Termini

**Figure 5:**
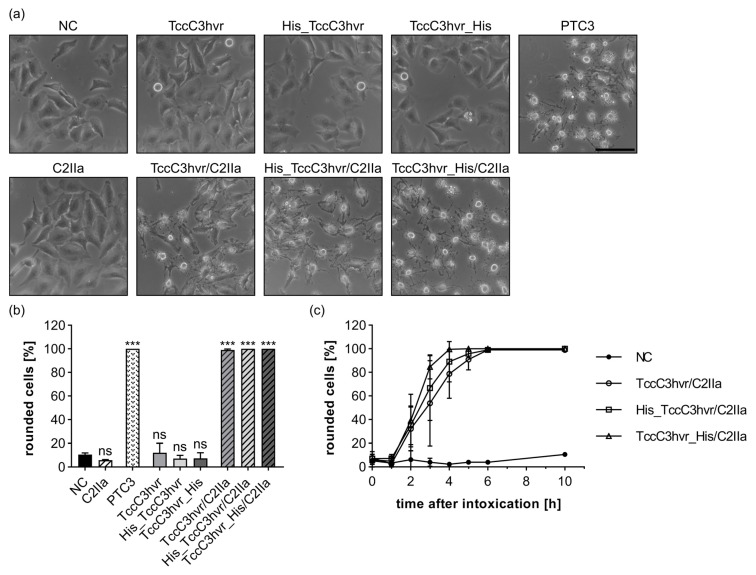
TccC3hvr constructs are efficiently transported into HeLa cells by C2IIa independent of a His-tag. (**a**) HeLa cells were treated as indicated with 10 nM C2IIa, 20 nM TccC3hvr cargo, PTC3 (1 µg/mL BC3 + 2 µg/mL TcdA1), or without toxin (NC). Representative pictures after 4 h incubation time are depicted. Scale bar corresponds to 100 µm. (**b**) The percentage of rounded cells from the same experiment as in (**a**) at 10 h after intoxication is shown. Values are given as mean ± SD (*n* = 3) of a triplicate from one representative experiment out of three replicates. Statistical analysis was performed compared to the NC by using non-parametric one-way ANOVA in combination with Dunnett’s correction for multiple comparison as described under Section 5.12 in Section 5 (ns *p* ≥ 0.05, *** *p* < 0.001). (**c**) Percentage of rounded cells from (**a**) at different time points. Values are given as mean ± SD (*n* = 3) of a triplicate from one representative experiment out of three replicates.

As a third cargo molecule, we tested the enzyme domain of the *Photorhabdus luminescens* toxin complex (amino acids 680–960). TccC3hvr is well suited as a cargo protein, since it specifically ADP-ribosylates F-actin, which causes characteristic membrane blebbing, breakdown of the cytoskeleton, and cell rounding [36,53]. Untagged (TccC3hvr), N-terminally His-tagged (His_TccC3hvr), and C-terminally His-tagged (TccC3hvr_His) versions of TccC3hvr were generated (for primary structures, see Supplementary Figure S1c). The purity and presence of the His-tags were confirmed for all TccC3hvr variants via SDS-PAGE and western blot (Supplementary Figure S2a,b). Moreover, all generated constructs were proven enzymatically active and were therefore suitable as reporter enzymes (Supplementary Figure S5). In contrast to the previous experiments, in combination with C2IIa, all TccC3hvr variants induced morphological changes similar to that of the wild type *Photorhabdus luminescens* toxin complex 3 (PTC3) (for phase contrast images, see Figure 5a; for quantification, see Figure 5b). Hence, the cytosolic release of TccC3hvr was found to be completely independent of the presence or position of the His-tag. Since these findings were quite unexpected, we confirmed them on another cell line, i.e., on Vero cells, with similar results (see Supplementary Figure S12). Nevertheless, our results indicate that the transport of untagged TccC3hvr is specifically dependent on C2IIa, since none of the TccC3hvr variants alone induced morphological changes on HeLa cells or Vero cells. The transport of the TccC3hvr variants via C2IIa could also be inhibited over a time course of 2–6 h via BafA1 or Quina as exemplarily tested for His_TccC3hvr. This suggests dependence on endosomal acidification and translocation through the C2IIa pore (for phase contrast images, see Supplementary Figure S13a; for quantification, see Supplementary Figure S13b; and for time course, see Supplementary Figure S13c). This transport via C2IIa was also confirmed in an established translocation assay, in which endosomal trafficking is blocked by keeping the cells on ice and incubation with BafA1. Translocation of the cargo protein through the plasma membrane is triggered via acidification of the warmed (37 °C) extracellular medium. This allows the investigation of the translocation step in an isolated manner. Only if the TccC3hvr variant was combined with C2IIa (exemplarily investigated for His_TccC3hvr/C2IIa) could cell rounding be detected after the acidic pulse (pH 4), while the single components His_TccC3hvr or C2IIa had no effects (for phase contrast images, see Supplementary Figure S13d; for quantification, see Supplementary Figure S13e). Moreover, the acidic pulse was required for His_TccC3hvr translocation, since treatment at pH 7.4 did not cause cell rounding. In conclusion, the transport of the TccC3hvr variants depends on C2IIa’s mode of action and translocation through the C2IIa pore.

Since the transport of untagged TccC3hvr via C2IIa was unexpected, we had a look at different features of the known C2IIa cargo proteins (His_DTA, His_TcdB-GTD, TccC3hvr, and C2I). Even though C2I and TccC3hvr are both ADP-ribosyltransferases with actin as an intracellular target, they share only 17% sequence identity, which is even lower when comparing the C2IIa-binding part C2IN with TccC3hvr (14%, calculated via BLOSUM62 [52]). Notably, the isoelectric point of DTA (theoretical pI 5.1) and TcdB-GTD (theoretical pI 4.7) is lower than that of TccC3hvr (theoretical pI 9.7). Hence, TccC3hvr is positively charged at neutral pH, while the net charge of DTA and TcdB-GTD is negative.

**Figure 6:**
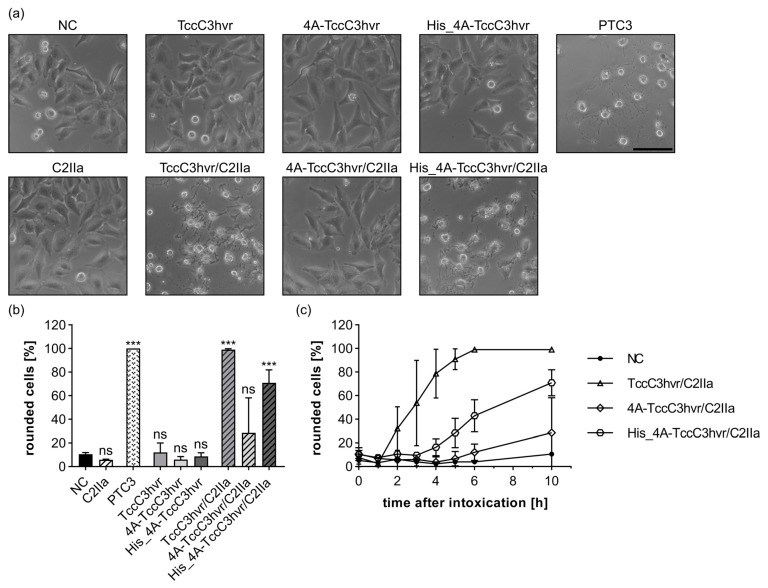
Transport of TccC3hvr by C2IIa is dependent on the positive charges at the N-terminus of TccC3hvr. (**a**) HeLa cells were treated as indicated with 10 nM C2IIa, 20 nM TccC3hvr cargo, PTC3 (1 µg/mL BC3 + 2 µg/mL TcdA1), or without toxin (NC). Representative pictures after 10 h incubation time are depicted. The experiment for the TccC3hvr constructs was performed in parallel to Figure 5, Figures S14 and S15. Scale bar corresponds to 100 µm. (**b**) The percentage of rounded cells from the same experiment as in (**a**) at 10 h after intoxication is shown. Values are given as mean ± SD (*n* = 3) of a triplicate from one representative experiment out of three replicates. Statistical analysis was performed compared to the NC by using non-parametric one-way ANOVA in combination with Dunnett’s correction for multiple comparison as described under Section 5.12 in Section 5 (ns *p* ≥ 0.05, *** *p* < 0.001). (**c**) Percentage of rounded cells from (**a**) at different time points. Values are given as mean ± SD (*n* = 3) of a triplicate from one representative experiment out of three replicates.

However, our results suggest that positive charges have to be positioned at the far N-terminal end for successful transport via C2IIa or PA_63_, since the N-terminal His-tags are more efficiently delivered into the cytosol compared to the C-terminal ones (Figure 1 and Figure 4). Therefore, we analyzed the primary structure of TccC3hvr and found four positively charged amino acids at its far N-terminal end (see Supplementary Figure S1c). To investigate whether this motif “PTIAE_5_ RIAAL_10_ KKNKV_15_” (positively charged amino acids marked in blue and negatively charged in red) within the first 15 amino acids is responsible for the transport of TccC3hvr via C2IIa, we exchanged all four positively charged amino acids against alanine residues (see Supplementary Figure S1c). The resulting mutated TccC3hvr variant is hereinafter designated as 4A-TccC3hvr. Even though 4A-TccC3hvr was still enzymatically active (Supplementary Figure S5a,b), this variant did not induce morphological changes in combination with C2IIa (for phase contrast images, see Figure 6a; for quantification after 10 h, see Figure 6b; for time course, see Figure 6c). These results strongly suggest that the positive charges at the N-terminal end of TccC3hvr are indeed responsible for the C2IIa-mediated delivery, and loss of the positive charges resulted in inefficient cytosolic delivery.

Additionally, we generated a rescue variant by fusing a 6xHis-tag at the N-terminus of 4A-TccC3hvr (His_4A-TccC3hvr) (for primary structure, see Supplementary Figure S1c). In combination with C2IIa, His_4A-TccC3hvr induced cell rounding in HeLa cells, which was not observed when the cells were treated with His_4A-TccC3hvr alone (for phase contrast images, see Figure 6a; for quantification after 10 h, see Figure 6b; for time course, see Figure 6c). Hence, the positively charged histidines at the N-terminus can recover the transportability via C2IIa into the cytosol of target cells. Comparable results for the transport of untagged TccC3hvr, 4A-TccC3hvr, and the rescue variant His_4A-TccC3hvr were obtained in combination with PA_63_ (for TccC3hvr/PA_63_, see Supplementary Figure S14; for 4A-TccC3hvr/PA_63_ or His_4A-TccC3hvr/PA_63_, see Supplementary Figure S15), indicating that C2IIa and PA_63_ share common features and that both are able to transport untagged proteins into the cytosol of target cells if the N-terminus is positively charged.

Taken together, C2IIa and PA_63_ can mediate the transport of various cargo molecules dependent on positive charges at the far N-terminal end of the protein.

## 3. Discussion

In modern pharmacology, protein- and peptide-based therapeutics play an important role in the treatment of many diseases, and their development is steadily increasing. Most of these so-called biologics are directed against extracellular targets, since usually they are unable to cross cellular membranes [54,55]. However, many important drug targets are located in the cytosol of cells, which significantly limits the therapeutic applications and potential of such molecules. Therefore, molecular transport systems for efficient delivery of therapeutic proteins and peptides are urgently needed to overcome this problem. Bacterial protein toxins should be the ideal candidates, since they are by nature designed to efficiently deliver enzymes, i.e., their A-subunits cross membranes into the cytosol of target cells (for reviews, see [55,56]). One of the best characterized molecules for this purpose is the anthrax toxin pore PA_63_, which has been used to deliver a large variety of cargo molecules into the cytosol of target cells (for review, see [27]). This platform is especially interesting for proteins that do not tolerate fusion with large adapter proteins, since PA_63_ can deliver proteins with a short N-terminal polyhistidine-, polyarginine-, or polylysine-tag which do not require a specific folding [35]. Since the B-subunit from the anthrax toxin is very similar in its structure and composition to the B-subunit of the C2 toxin from *C. botulinum* (C2IIa) [2,13], we investigated whether C2IIa could function as molecular Trojan horse for cytosolic delivery of His-tagged cargo molecules.

In the present study, we demonstrated that C2IIa can be used as a protein delivery tool for transporting N-terminally His-tagged enzymes into the cytosol of human target cells. Hence, C2IIa and PA_63_ share a conserved mechanism of translocation and similar pore features for interacting with positively charged proteins. Even though the exact binding motifs in C2IIa or PA_63_ for positively charged tags still have to be identified, it has been speculated by others that negatively charged amino acids in the vestibule of the PA_63_ pore play a crucial role for this interaction [38]. Our dot blot experiment and STED-super resolution microscopic data indicate that C2IIa directly interacts with His-tagged cargo proteins, which strongly enhances their uptake into early endosomes. Therefore, we can exclude that His-tagged proteins directly interact with the plasma membrane and are simultaneously taken up together with C2IIa, as discussed for PA_63_ by Blanke et al. [35]. Moreover, for uptake and translocation of the His-tagged cargo proteins, the mode of action of C2IIa is exploited. The delivery of the His-tagged proteins via C2IIa was successfully inhibited by the V-ATPase inhibitor BafA1 [5,48] and the C2IIa pore blocker Quina [46,47], demonstrating the dependency on endosomal acidification and protein translocation through the C2IIa pore. Schleberger et al. suggested that under low pH conditions in the endosome, the N-terminal Helix α1 of C2I possesses a positive net charge and could form a bolt that initiates translocation through the C2IIa pore [13]. Potentially, the His-tag could imitate this positively charged bolt and trigger the protein translocation process. However, not all His-tagged proteins are equally efficiently delivered via C2IIa or PA_63_ into the cytosol of target cells. We found that the position of the His-tag plays a crucial role in the transport via C2IIa or PA_63_. C-terminally His-tagged proteins were considerably less efficiently delivered compared to N-terminal ones. These results emphasize that, for efficient transport, the correct orientation of the cargo protein is essential, and similar to the translocation model of the native cargo C2I [13], the N-terminal end has to cross the pore first. To the best of our knowledge, this dependence on the position of the polycationic tag was also shown for the first time for the PA_63_-mediated transport. However, as shown for His_eGFP_DTA, not all N-terminally His-tagged proteins are efficiently delivered into the cytosol via PA_63_ or C2IIa. Blanke et al. showed that by using longer polylysine-tags (3x < 6x < 8x), the transport efficiency was significantly enhanced [35]. Even with a decahistidine-tag used for His_eGFP_DTA, transport of this cargo molecule did not take place efficiently (for primary structures, see Supplementary Figure S1a). One important prerequisite for the translocation of cargo through the C2IIa or PA_63_ pores is that cargo molecules have to at least partially unfold and refold [13,57,58]. Most probably, the endosomal translocation process of His_eGFP_DTA via C2IIa or PA_63_ is blocked, due to the stable β-barrel fold of eGFP. At first glance, this hypothesis seems to be contradictory to the published results of Zornetta et al., who showed that fusion proteins based on the anthrax toxin’s A-subunits and eGFP (LF_eGFP and EF_eGFP) are delivered into the cytosol of target cells [49]. However, as shown in their intoxication assays, the cytosolic release of these cargo molecules was strongly reduced by eGFP-labeling [49], which could be explained by our findings. Moreover, degradation of their fusion proteins cannot be excluded, and delivery of only the truncated EF- or LF-part without the eGFP-label would cause false-positive results. In contrast, in our assays, false-positive results caused by degradation of the fusion proteins can be excluded due to the position of DTA in the construct. Cells can only be intoxicated by His_eGFP_DTA if the N-terminal His-tag, which is essential for the transport via C2IIa or PA_63_ pore as well as the C-terminal DTA used as the reporter enzyme, are present together. Hence, only transport of the complete fusion construct into the cytosol will be detected in our intoxication assays. As shown via STED super resolution microscopy, C2IIa facilitated endocytosis of His_eGFP_DTA, indicating that interaction of C2IIa and His_eGFP_DTA took place and mediated the uptake into endosomes. Most probably, the translocation step was blocked by eGFP in our assays. Taken together, our results indicate that eGFP does not or only inefficiently translocate through the C2IIa or PA_63_ transmembrane pores in cells, which is most probably caused by insufficient unfolding of eGFP inside endosomes.

By using TccC3hvr as a cargo molecule, we found that untagged proteins with a naturally positively charged N-terminus can be delivered via the C2IIa or PA_63_ machinery into the cytosol of target cells. At neutral pH, four positively charged amino acids and one negatively charged amino acid were present in the first 15 residues of TccC3hvr. Hence, a net charge of 3+ could be sufficient for the transport via C2IIa or PA_63_. This is consistent with the results obtained by Blanke et al., who showed that a 3x polylysine-tag at the N-terminus of DTA could mediate transport via PA_63_ [35]. A notable difference is that in the case of the TccC3hvr, the positive amino acids were separated by uncharged residues. These results are interesting for using C2IIa or PA_63_ as delivery platforms, since proteins or peptides with a positively charged N-terminus can be transported directly without addition of a His-tag.

Despite all these similarities found between C2IIa and PA_63_, there are also some important differences. While Lys_DTA was about 100-fold more efficiently delivered into cells via PA_63_ compared to His_DTA, this was not the case for C2IIa. In contrast, C2IIa preferred His_DTA over Lys_DTA as a cargo molecule. The reason for this unexpected behavior of C2IIa is unknown. We hypothesize that there might be an optimum of positive charges for the N-terminus of cargo proteins that should not be exceeded for efficient C2IIa delivery. The pK_a_ value of histidine (pK_a_ 6.04) is lower compared to that of lysine (pK_a_ 10.67) or arginine (pK_a_ 12.10) [59], implying that at neutral pH less than half of the histidines in a 6x His-tag are protonated and positively charged, while most of the lysine residues are positively charged in the 6x Lys-tag. Potentially, more positive charges could increase the affinity of the cargo molecule to C2IIa. However, due to this stronger interaction of C2IIa and the cargo protein, translocation or cytosolic release of the molecule might be blocked. Interestingly, for PA_63_, it was found that 6x Lys-tags are 100-fold more efficiently delivered than 6x Arg-tags, which was explained by the degradation of the 6x Arg-tag [35]. Alternatively, one could speculate that also for PA_63_, there is an optimal number of positive charges which is higher than that of C2IIa, but this optimum was also exceeded with Arg-tagged cargo molecules. Following this hypothesis, once the optimal charge for C2IIa or PA_63_-mediated transport is exceeded, cytosolic release becomes less efficient. Nevertheless, further mechanistic investigations are needed to support this hypothesis. Moreover, it could be speculated that a certain combination of positively charged residues potentially enhances the C2IIa- or PA_63_-mediated translocation further. This is plausible, since TccC3hvr is still more efficiently transported into the cytosol compared to the rescue variant His_4A-TccC3hvr, indicating that the His-tag could not restore the full transport capability of the four positively charged amino acids present in TccC3hvr. However, the optimal polycationic-tag for PA_63_- or C2IIa-mediated delivery still has to be identified in future studies.

Another structural difference between C2IIa and PA_63_ is their inner pore diameter. The inner pore diameter of PA_63_ is 6 Å at the narrowest part (Φ clamp) [60], while the transmembrane pore of C2IIa possesses a minimal inner diameter of 27 Å [13]. Thereby, the PA_63_ pore is so narrow that proteins most likely have to unfold completely (as do their secondary structures) for translocation [60]. Contrary to that, for C2IIa-mediated transport, cargo proteins have to be at least partially unfolded for translocation [13]. Potentially, this structural difference could have a big impact on cargo delivery if complete unfolding of a protein is not reversible or if larger protein modifications are attached to the cargo. In these cases, C2IIa could be a valuable alternative for delivery of His-tagged cargo into the cytosol of target cells, in particular as C2IIa targets all cell types.

## 4. Conclusions

Taken together, we demonstrated that C2IIa can serve as a molecular Trojan horse for the delivery of N-terminally His-tagged proteins or untagged proteins with a positively charged N-termini into the cytosol of target cells. Thereby, protein delivery depends on charge strength, position of the positive charges, as well as on the ability of the cargo protein to unfold at least partially for translocation through the C2IIa pore. Our findings pave the way for novel cell biological or pharmacological applications of C2IIa as a protein delivery tool and show that C2IIa shares some basic features in the protein translocation mechanism with the anthrax toxin pore PA_63_.

## 5. Materials and Methods

### 5.1. Molecular Cloning and Mutagenesis

Multiple His-, Lys-, or GST-tagged constructs were generated (Supplementary Figure S1). Untagged proteins were generated by proteolytic removal of the GST-tag after protein purification (see Section 5.4).

Short polypeptide tags were deleted or inserted via inverse polymerase chain reaction (PCR) site-directed mutagenesis. PCRs were performed with Q5 High-Fidelity DNA Polymerase (New England Biolabs, Ipswich, MA, USA) according to the manufacturer’s protocol. For site-directed mutagenesis, the modified blunt-end PCR-products (5% *v*/*v*) were phosphorylated and ligated in 1-fold T4 DNA ligase buffer by incubation with 0.125 units/μL T4 polynucleotide kinase and 20 units/μL T4 DNA ligase (all from New England Biolabs, Ipswich, MA, USA) for 1 h at room temperature (RT). Methylated template DNA was digested by addition of 0.2 units/μL Dpn I (Thermo Fisher Scientific, Waltham, MA, USA) and further incubation for 1 h at 37 °C.

For the generation of GST-tagged fusion proteins, traditional cloning techniques were used. Inserts were amplified by overhang-primer PCR, and the restriction enzymes BamHI and EcoRI (all from New England Biolabs, Ipswich, MA, USA) were used for generation of sticky ends. Digested target vectors and inserts were mixed in different ratios (1:3, 1:7, or 1:10) and ligated with T4 DNA ligase according to the manufacturer’s protocol.

After heat-shock transformation, the generated plasmids were amplified in *Escherichia* (*E.*) *coli* DH5α (Clontech Laboratories, Inc., Heidelberg, Germany). Plasmids were purified according to the my-Budget plasmid mini kit (Bio-Budget Technologies GmbH, Krefeld, Germany) protocol. The correct sequence of the plasmid’s open reading frames was verified using Lightrun Sanger sequencing (Eurofins Genomics, Luxemburg, Luxemburg).

### 5.2. Recombinant Protein Expression and Cell Lysis

For recombinant protein expression, *E. coli* BL21 (Novagene Madison, WI, USA) cells were heat-shock transformed with the verified plasmids. A single colony of transformed *E. coli* cells was used for inoculation of the first preculture in 5 mL LB-medium (1% tryptone, 0.5% yeast extract, 1% NaCl, 100 µg/mL ampicillin), which was cultured for 5–8 h at 37 °C and 180 rpm in a shaking incubator (Benchmark Scientific, Sayreville, NJ, USA). The complete volume of the first preculture was used to inoculate a second 150 mL overnight-preculture in LB-medium incubated at 37 °C and 180 rpm. Of this overnight preculture, 120 mL was used to inoculate the main culture in 4 L LB-medium. The main culture was incubated at 37 °C until an OD_600_ of 0.6–0.8 was reached. Subsequently, protein expression was induced with 0.5 mM isopropyl-d-1-thiogalactopyranoside (IPTG, Carl Roth, Karlsruhe, Germany). After an incubation at 37 °C, 180 rpm for 3 h (DTA and TccC3hvr constructs) or at 29 °C and 180 rpm overnight (~18 h) (TcdB-GTD constructs), the *E. coli* cells were harvested by centrifugation at 5500 rcf and 4 °C for 10 min. For His-tagged proteins, the cells were resuspended in 40 mL NPI-20 (50 mM NaH_2_PO_4_, 300 mM NaCl, 20 mM imidazole, pH 8.0), and 1% phenylmethylsulfonyl fluoride (PMSF) was added, while 40 mL lysis-buffer (10 mM NaCl, 20 mM Tris, 1% Triton X-100, 1% PMSF) was used for GST-tagged proteins. Cells were lysed by sonication (10 × 30 s pulses and intermediate pauses of 30 s), and the insoluble cell fragments and remaining cells were removed by centrifugation at 13,000 rcf at 4 °C for 30 min. The supernatant was filtered with 0.45 µm and 0.2 µm syringe filters.

### 5.3. Purification of His-Tagged Proteins

His-tagged proteins were either purified using a batch purification protocol (TccC3hvr constructs) or with a FPLC purification protocol (DTA and TcdB-GTD constructs).

For the batch purification protocol, 1 mL bed-volume of PureCube 100 INDIGO Ni-agarose (Cube Biotech, Monheim am Rhein, Germany) was equilibrated with NPI-20 and then incubated overnight with the filtered cell lysate at 4 °C in an overhead shaker. For protein elution, Midi Plus columns (Cube Biotech, Monheim am Rhein, Germany) were used according to the manufacturer’s instructions at 4 °C. NPI-20 was used as equilibration and washing buffer. NPI-250 (50 mM NaH_2_PO_4_, 300 mM NaCl, 250 mM imidazole, pH 8.0) was used as elution buffer with 6 steps of 1 mL followed by 2 steps of 0.5 mL.

For the FPLC purification protocol, an ÄKTA go protein purification system (Cytiva, Marlborough, MA, USA) was used at 4 °C. Protino Ni-NTA 1 mL FPLC columns (MACHEREY-NAGEL, Düren, Germany) were used with a flow rate of 1 mL/min. After equilibration with 5 mL of NPI-20, the filtered cell lysate was loaded onto the column. The column was washed with 15 mL NPI-20, followed by a fractionated elution (each 0.5 mL) with 10 mL of NPI-250 in total.

For both purification protocols, fractions were analyzed using SDS-PAGE and Coomassie staining. The fractions with high concentration and purity were pooled, and the volume was reduced using Vivaspin 20 (Sartorius, Göttingen, Germany) centrifugation columns (MWCO smaller than a third of the size of the purified protein). Dialysis (Spectra/Por^®^6, Spectrum Laboratories, Rockleigh, NJ, USA) was performed overnight at 4 °C to rebuffer the purified proteins into PBS with a dilution greater than 1:1000. The concentration of the purified proteins was determined against a BSA standard with densitometric analysis after SDS-PAGE with subsequent Coomassie staining.

C2IIa was activated from purified His_C2II as described earlier [47].

### 5.4. Purification of Proteins via GST-Tag and Tag Removal

The sterile-filtered cell lysates were incubated at 4 °C overnight with 1.2 mL Protino Glutathione Agarose 4B-beads (Macherey-Nagel, Düren, Germany) equilibrated in PBS (137 mM NaCl, 2.7 mM KCl, 8 mM Na_2_HPO_4_, and 1.8 mM KH_2_PO_4_; pH 7.4). The beads were washed twice with washing buffer (150 mM NaCl, 20 mM Tris HCl; pH 7,4) and once with PBS by centrifugation at 3000 rcf for 5 min. For cleavage of the GST-tag and elution of the protein, the beads were incubated for 1 h at room temperature with 30 NIH units of thrombin (Amersham Biosciences, Little Chalfont, UK) per liter of main bacterial culture. Following centrifugation at 10,000 rcf for 30 s at 4 °C, the supernatant was transferred onto 45 µL Benzamidine–Sepharose 6B-beads (GE Healthcare, Chicago, IL, USA) and incubated at room temperature for 10 min to deplete the thrombin. Centrifugation at 10,000 rcf for 30 sec at 4 °C removed the benzamidine beads. The purified protein concentration was determined as mentioned above.

### 5.5. Cell Culture

For cultivation of HeLa cells and Vero cells, MEM medium (Gibco-Life Technologies, Carlsbad, CA, USA) supplemented with 10% fetal calf serum (Gibco-Life Technologies, Carlsbad, CA, USA), 1% sodium pyruvate (Gibco-Life Technologies, Carlsbad, CA, USA), 1% L-Glutamin (PAN-BIOTECH, Aidenbach, GER), 1% MEM-NEAA (Gibco-Life Technologies, Carlsbad, CA, USA), and 100 U/mL (1%) penicillin–streptomycin (Gibco-Life Technologies, Carlsbad, CA, USA) was used. HeLa cells and Vero cells were cultured at 37 °C, 5% CO_2_ with constant humidity and subcultivated every 3 to 4 days with a split ratio of 1:3 to 1:10 after trypsinization (PAN-BIOTECH, Aidenbach, Germany).

### 5.6. Phase-Contrast Microscopy

Cells were seeded in microtiter plates, treated as indicated, and one image per well was recorded with a phase contrast microscope (Leica microsystems, Wetzlar, Germany). Representative images with scale bars are shown. Cells were quantified by semi-automatic cell image analysis via neuralab.de software (Neuralab, Ulm, Germany), and each analysis was verified manually.

### 5.7. STED Super-Resolution Microscopy

STED-microscopy was performed as described in [61,62]. A total of 1.8 × 10^5^ HeLa cells were seeded per well in an 8-well µ-slide with glass bottom (ibidi GmbH, Gräfelfing, Germany). The cells were treated with the indicated proteins and concentrations (250 nM for His_eGFP_DTA and 25 nM for C2IIa) for 30 min at 37 °C. Subsequently, the cells were washed twice with PBS. For fixation, the samples were treated for 20 min at RT with 3.2% paraformaldehyde in PBS (32% PFA aqueous solution, Electron Microscopy Sciences, Hatfield, PA, USA). The cells were washed three times with PBS, permeabilized, and blocked by incubation with 3% BSA and 0.3% TritonX-100 in PBS for 2 h at RT. For immunohistological staining, the cells were incubated at 4 °C overnight with 1 µg/mL of primary rabbit anti-EEA1 antibody (Thermo Scientific, Waltham, MA, USA) and 0.5 µg/mL Atto594-conjugated GFP-booster nanobody (Chromotek, Planegg Germany) in 1:10 diluted permeabilization/blocking solution. After three washing steps, samples were incubated with 1 µg/mL of the secondary Atto647N-conjugated goat anti-rabbit antibody (Sigma-Aldrich, St. Louis, MO, USA) dissolved in 1:10 diluted permeabilization/blocking solution for 1 h at RT. Three additional washing steps with PBS were used for removal of unbound antibodies, and PBS was exchanged with 2,2′-thiodiethanol (97% solution in PBS, pH 7.5) before imaging.

The samples were analyzed with a home-built dual-color 3D STED microscope [63]. Images were recorded with an average power of 0.8 µW for the excitation beams, 1.3 mW for depletion beams, a dwell time of 300 µs, a typical peak photon number of approximately 150 counts, and a pixel size of 12.5 nm. For image analysis of the recorded pictures, ImageJ (v1.52n, National Institute of Health, Bethesda, MD, USA) was used. For better visualization, a σ = 1 pixel Gaussian blur and >20 count intensity threshold were applied. A self-written search algorithm (Python 3.7, available on request) was used to count the eGFP signals, as described earlier [61], and a threshold of 10 counts was applied. For each experiment, the mean number of eGFP signals was calculated and averaged for five individual experiments (*n* = 5).

### 5.8. Cell Viability/Proliferation Assay

A total of 7.7 × 10^3^ cells/well were seeded in 96-well microtiter plates one day before treatment and treated as indicated. Subsequently, 10% CellTiter 96^®^ AQueous One solution containing 3-(4,5-dimethylthiazol-2-yl)-5-(3-carboxymethoxyphenyl)-2-(4-sulfophenyl)-2Htetrazolium (MTS) (Promega, Madison, WI, USA) was added and incubated for 1–2 h before measuring the absorbance at 492 nm in a TriStar^2^ LB 942 microplate reader (Berthold Technologies GmbH & Co.KG, Bad Wildbad, Germany).

### 5.9. OPP-Based Protein Synthesis Assay

HeLa cells were seeded in 24-well microtiter plates (10^5^ cells/well), incubated for 24 h (37 °C and 5% CO_2_), and treated with toxins as indicated. The Click-&-Go^TM^ Plus OPP Protein Synthesis Assay Kit (Click Chemistry Tools, Scottsdale, AZ, USA) was used to monitor changes in protein synthesis. The assay was performed as described in the manufacturer’s protocol, and deviations are indicated below. After incubation of the cells with 5 µM OPP-reagent diluted in culture medium for 30 min, cells were scratched off the plate in their respective intoxication medium containing 5 µM OPP, transferred into 1.5 mL reaction tubes, and centrifuged at 500 rcf for 5 min (washing step). The supernatant was removed, and the cells were washed with 1% BSA (Carl Roth, Karlsruhe, Germany) in PBS. The cells were fixed for 15 min with 4% paraformaldehyde (Merck, Darmstadt, Germany) in PBS at RT, and the washing step was repeated. Permeabilization was performed with a saponin-based (Sigma-Aldrich, St. Luis, MO, USA) washing reagent (0.1% saponin, 0.5% BSA, 0.01 NaN_3_) for 15 min at RT, followed by washing. After removal of the supernatant, 100 µL Reaction Cocktail was added to each sample and incubated in the dark for 20 min at RT, followed by another washing step (1% BSA in PBS, 500 rcf, 5 min). Eventually, the cells were re-suspended in PBS, and fluorescence of the cells was measured by flow cytometry with a BD FACSCelesta^TM^ Cell Analyzer (Becton, Dickinson and Company, Franklin Lakes, NJ, USA). Data were analyzed with Flowing Software 2.5.1 (Turku Bioscience, Turku, Finland).

### 5.10. SDS-PAGE and Western Blot

For electrophoresis, SDS-PAGE using a 12.5% acrylamide gel was performed. After separation of the proteins by their molecular weight, the gel was either stained with Coomassie Brilliant Blue R250 (SERVA Electrophoresis GmbH, Heidelberg, Germany) or the proteins were transferred onto a nitrocellulose membrane by semi-dry western blotting. The transfer was controlled using Ponceau S (AppliChem GmbH, Darmstadt, Germany) staining. The membrane was then blocked in 5% skim milk powder solution in PBS-T (137 mM NaCl, 2.7 mM KCl, 8 mM Na_2_HPO_4_, 1.8 mM KH_2_PO_4_, 0.1% Tween20; pH 7.4) for 1 h at room temperature (RT), followed by a washing step with PBS-T. The membrane was either incubated for 1 h at RT with streptavidin-peroxidase conjugate (1:5000 in PBS-T; Sigma-Aldrich, St. Louis, MO, USA) or overnight at 4 °C with the indicated antibodies diluted in PBS-T. The loading controls Hsp90 and GAPDH were detected with 1:1000 diluted mouse Hsp90 α/β antibody (F-8, Santa Cruz Biotechnology, Dallas, TX, USA) or 1:2000 diluted mouse GAPDH antibody (G-9, Santa Cruz Biotechnology, Dallas, TX, USA), respectively. Rac1 was detected with a 1:500 diluted mouse anti-non-glucosylated Rac1 antibody (102/Rac1, 1:500, BD Biosciences, San Jose, CA, USA). DTA- and TcdB-containing constructs were each detected using a 1:1000 dilution of mouse anti-diphtheria toxin A antibody (8G2, Bio-Rad Laboratories, Hercules, CA, USA) or rabbit anti-*Clostridioides difficile* Toxin B antibody (ERP23357-19, Abcam plc, Cambridge, UK). His-tagged proteins were detected by 1:2000 diluted mouse 6x His-tag monoclonal antibody (HIS.H8, Thermo Fisher Scientific, Waltham, MA, USA). After washing the membrane three times for 5 min with PBS-T, it was incubated for 1 h at RT with the respective 1:2500 diluted, secondary antibody Goat anti-Mouse IgG (H + L) secondary antibody, horse-reddish peroxidase (HRP) (Thermo Fisher Scientific, Waltham, MA, USA), mouse IgG kappa binding protein-HRP (Santa Cruz Biotechnology, Dallas, TX, USA), or mouse anti-rabbit IgG-HRP (Santa Cruz Biotechnology, Dallas, TX, USA). Unbound antibodies were removed by washing with PBS-T three times. HRP-conjugated antibodies or binding proteins were detected with Pierce ECL western blotting substrate (Thermo Fisher Scientific, Waltham, MA, USA) and X-ray films (AGFA Health Care, Mortsel, Belgium).

### 5.11. Probing of Intracellular Rac1-Glucosylation Status in Intact Cells

A total of 8 × 10^3^ cells per well were seeded for 2 days prior to intoxication in a 48-well plate. Cells were intoxicated as indicated. After intoxication, microtiter plates were transferred to ice and cooled down, and cells were mechanically detached and transferred into a reaction tube. Cells were centrifuged at 10,000 rcf for 30 s at 4 °C, and the pellet was washed once with PBS, centrifuged again, and resuspended in 20 µL PBS. Laemmli buffer (0.3 M Tris-HCl, 10% SDS, 37.5% glycerol, 0.4 mM bromophenol blue, 100 mM DTT) was added, samples were heat-denatured at 95 °C for 10 min and subjected to SDS-PAGE and western blotting. The non-glucosylated Rac1 signal of the western blot was normalized to the Hsp90 loading control and given as a percentage of the negative control.

### 5.12. Statistics

If not stated differently, all experiments were performed with at least three independent experiments. Individual replicates (*n*) were used for statistical analysis as indicated. For statistical analysis, a non-parametric one-way ANOVA was performed with Dunnett’s multiple comparison test (GraphPad, Version 9.5.1) as indicated. *p* values were indicated as following: not significant (ns) *p* ≥ 0.05; * *p* < 0.05; ** *p* < 0.01; *** *p* < 0.001.

## Data Availability

The data presented in this study are available on request from the corresponding author.

## Supplementary Materials

The following supporting information can be downloaded at https://www.mdpi.com/article/10.3390/toxins15060390/s1, Figure S1: Schematic representation of the primary structures of all protein constructs used as model cargo; Figure S2: Coomassie staining and Western Blot analysis of all used cargo proteins; Figure S3: All DTA constructs are enzymatically active; Figure S4: All TcdB-GTD constructs are enzymatically active; Figure S5: All TccC3hvr constructs are enzymatically active; Figure S6: Cytosolic release of His_DTA is dependent on the C2IIa concentration; Figure S7: Lys_DTA is transported more efficiently into HeLa cells than His_DTA by PA_63_; Figure S8: DTA variants only bind to C2IIa if a polycationic tag is present; Figure S9: C2IIa mediates the uptake of His_eGFP_DTA into early endosomes; Figure S10: TcdB-GTD is efficiently transported into HeLa cells by PA_63_ in the presence of an N-terminal His-tag; Figure S11: His_TcdB-GTD transport by C2IIa is inhibited by established inhibitors of C2IIa-mediated translocation; Figure S12: TccC3hvr constructs are transported into Vero cells by C2IIa; Figure S13: His_TccC3hvr transport by C2IIa is inhibited by established inhibitors of C2IIa-mediated translocation; Figure S14: TccC3hvr constructs are efficiently transported into HeLa cells by PA_63_ independent of a His-tag; Figure S15: Transport of TccC3hvr by PA_63_ is dependent on the positive charge at the N-terminus of TccC3hvr.
