# Supplementary material for: The Clostridium botulinum C2 Toxin Subunit C2IIa Delivers Enzymes with Positively Charged N-Termini into the Cytosol of Target Cells
Source: Toxins (Basel). 2023 Jun 9;15(6):390. doi: 10.3390/toxins15060390 (PMC10305195; doi:10.3390/toxins15060390)
Supplement: Supplementary file 1 [file toxins-15-00390-s001.zip › toxins-2417529-supplementary.pdf]

1. Supplementary Results

1.1. Primary Structure and Composition of Used Linkers and N- or C-Terminal Polycationic Tags

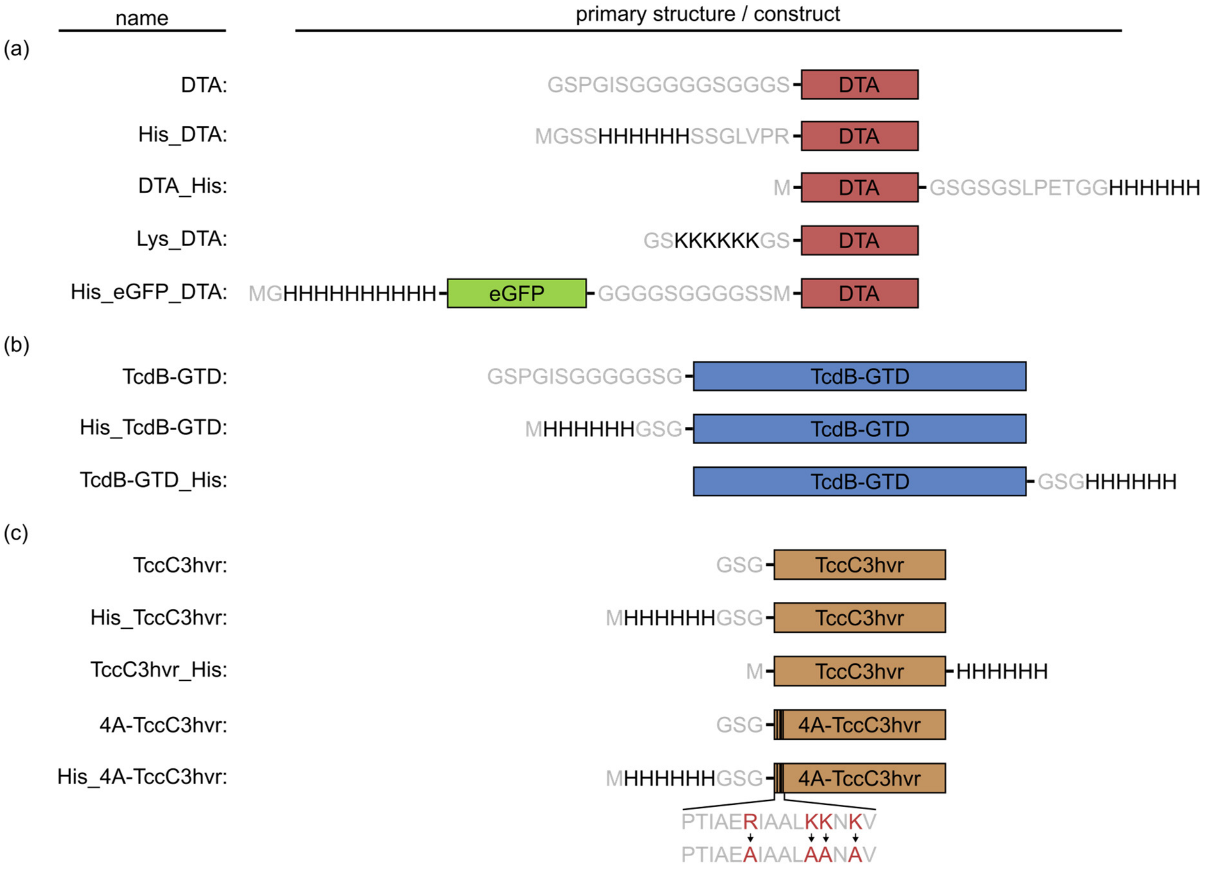

Figure S1. Schematic representation of the primary structures of all protein constructs used as model cargo. Primary structures of the reporters DTA, eGFP, TcdB-GTD and TccC3hvr are abbreviated with boxes. Polyhistidine- and polylysine-tags are indicated with black letters while other amino acids are depicted in gray. **(a)** DTA constructs with DTA (Uniprot P00588, amino acids 33-225) and eGFP (Uniprot C5MKY7, amino acids 2-239). **(b)** TcdB-GTD constructs with TcdB-GTD (Uniprot P18177, amino acids 1-543). **(c)** TccC3hvr constructs with TccC3hvr (Uniprot Q8GF97, amino acids 680-960). The first 15 residues of TccC3hvr and the 4A-TccC3hvr are indicated below the boxes for the wild type. Positively charged amino acids and the corresponding mutations in the 4A-TccC3hvr variant are marked in red. The upper sequence corresponds to the primary structure of (wild type) TccC3hvr while the lower sequence corresponds to the 4A-TccC3hvr mutant.

1.2. The Identity and Purity of the Generated Constructs Was Validated in Western Blot and SDS-PAGE

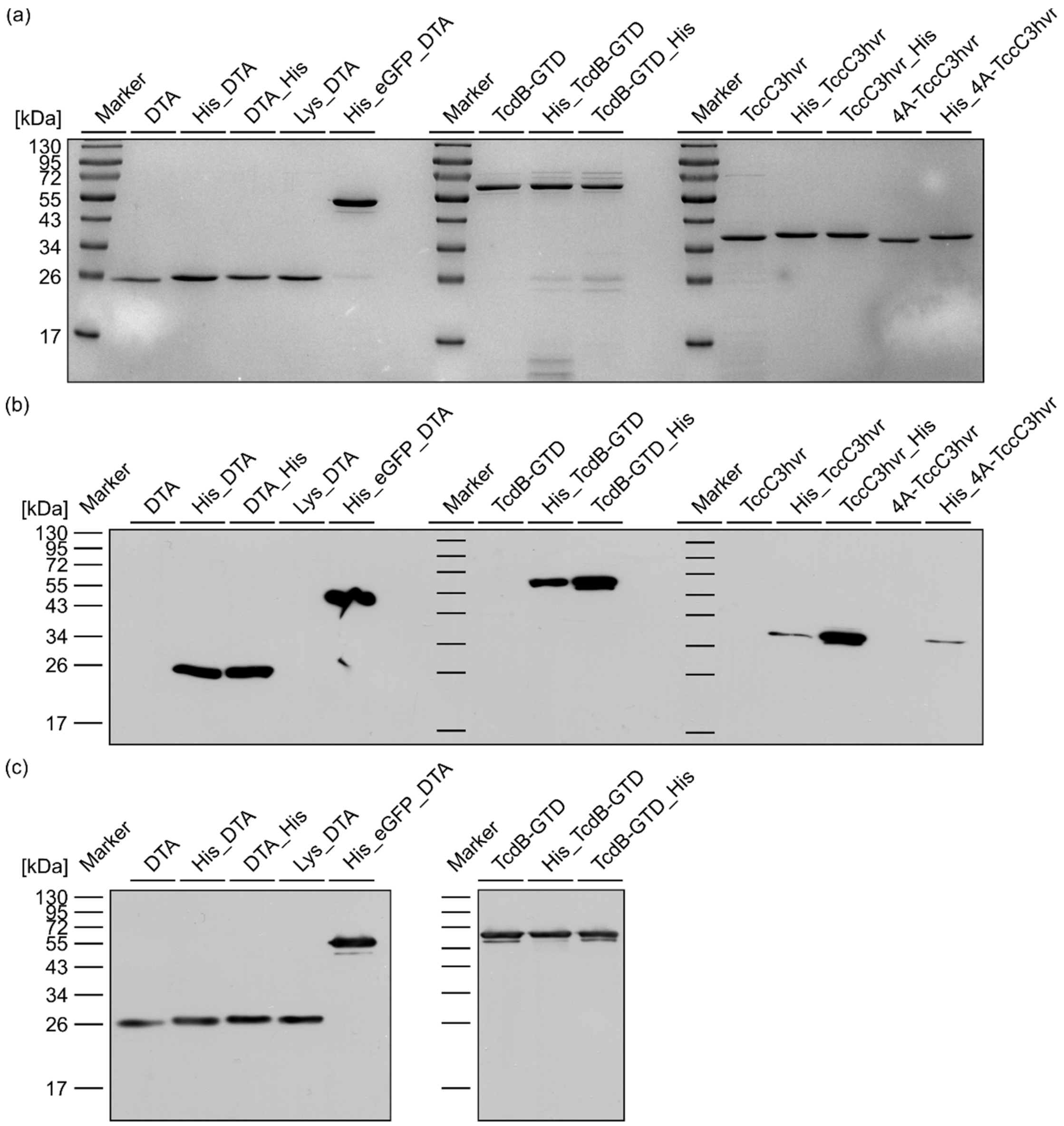

Figure S2. Coomassie staining and Western Blot analysis of all used cargo proteins. **(a)** 1 µg of each cargo protein was separated by SDS-PAGE and proteins were Coomassie stained to check for protein purity. **(b)** 10 pmol of each cargo protein were separated by SDS-PAGE and transferred to a nitrocellulose membrane by subsequent Western Blotting. Detection of His-tags was performed for all constructs. **(c)** 10 pmol of each cargo protein were separated by SDS-PAGE and transferred to a nitrocellulose membrane by subsequent Western Blotting. DTA (left blot) and TcdB-GTD (right blot) were detected respectively.

1.3. The Reporter Enzymes (DTA, TcdB-GTD, and TccC3hvr) Are All Enzymatically Active Independent of the Used Poly-cationic Tag and Its Position

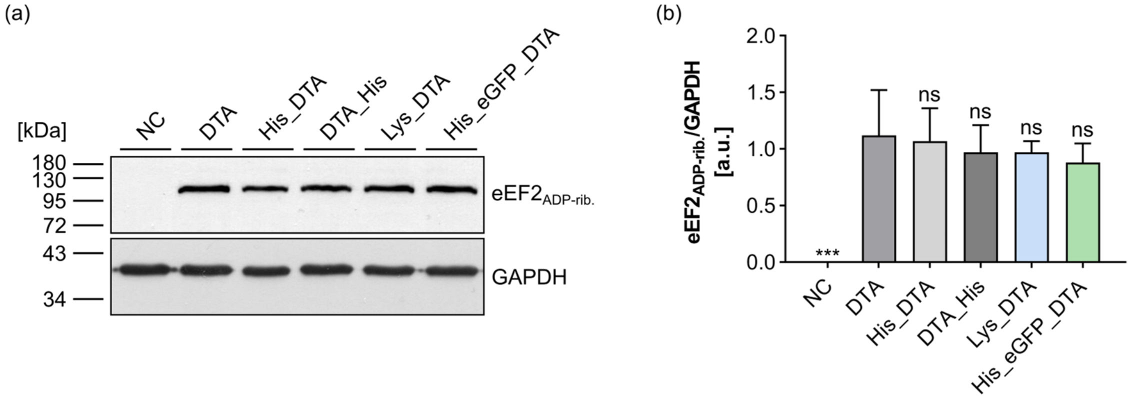

Figure S3. All DTA constructs are enzymatically active. **(a)** Representative Western Blot of an *in vitro* ADP-ribosylation assay of eEF2 by DTA constructs. **(b)** Quantification of the Western Blot from (a). The signal for ADP-ribosylated eEF2 was normalized to GAPDH loading control and normalized to the mean intensity of all DTA constructs. Values are given as mean ± SD (n = 6) of six individual sets from two experiments. Statistical analysis was performed compared to the incubation with DTA by using non-parametric one-way ANOVA in combination with Dunnett's correction for multiple comparison as described under Section 5.12. in the Materials and Methods section (ns $p \geq 0.05$, *** $p < 0.001$).

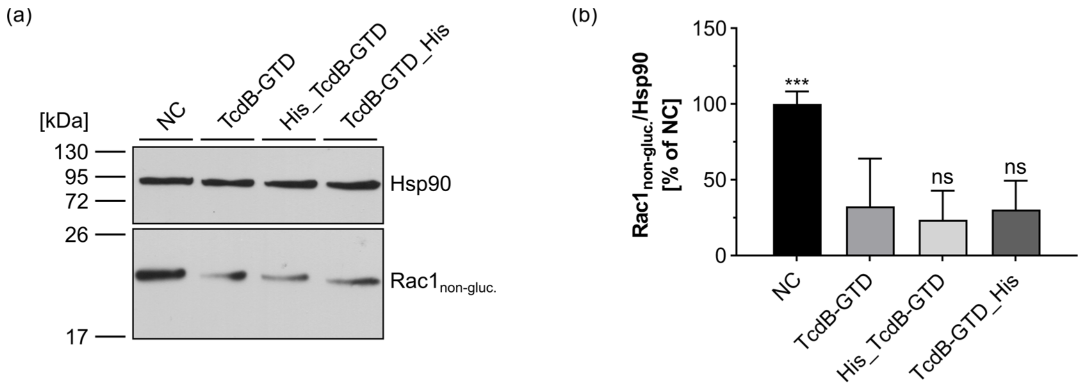

Figure S4. All TcdB-GTD constructs are enzymatically active. **(a)** Representative Western Blot of an in vitro glucosylation of Rac1 by TcdB-GTD constructs. (**b**) Quantification of the Western Blot from (a). The signal for Non-glucosylated Rac1 was normalized to Hsp90 loading control and given as percentage of NC. Values are given as mean ± SD (n = 6) of six individual sets from two experiments. Statistical analysis was performed compared to the incubation with TcdB-GTD by using non-parametric one-way ANOVA in combination with Dunnett's correction for multiple comparison as described under Section 5.12. in the Materials and Methods section (ns $p \geq 0.05$, *** $p < 0.001$).

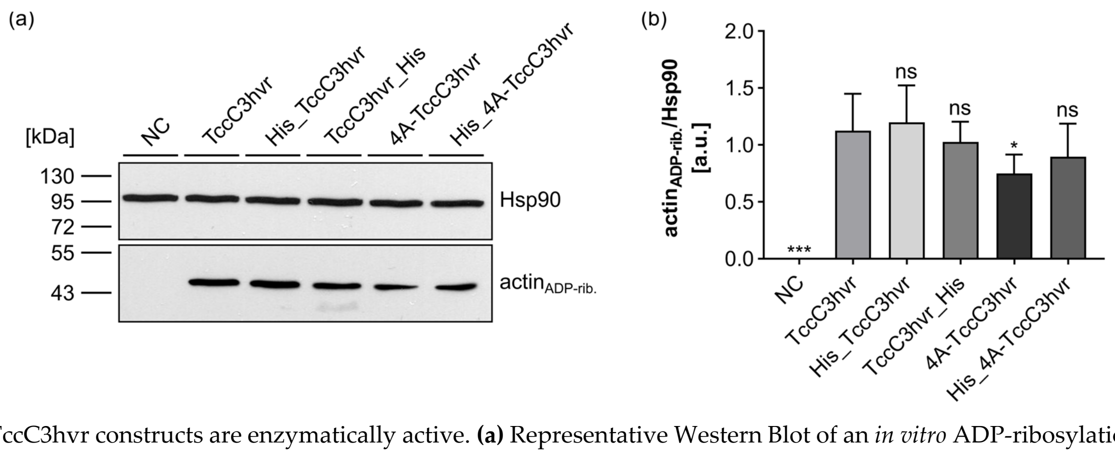

Figure S5. All TccC3hvr constructs are enzymatically active. **(a)** Representative Western Blot of an *in vitro* ADP-ribosylation assay of actin by TccC3hvr constructs. **(b)** Quantification of the Western Blot from (a). The signal for ADP-ribosylated actin was normalized to Hsp90 loading control and normalized to the mean intensity of all TccC3hvr constructs. Values are given as mean ± SD (n = 6) of six individual sets from two experiments. Statistical analysis was performed compared to the incubation with TccC3hvr by using non-parametric one-way ANOVA in combination with Dunnett's correction for multiple comparison as described under Section 5.12. in the Materials and Methods section (ns $p \geq 0.05$, * $p < 0.05$, *** $p < 0.001$).

1.4. *Cytosolic Delivery of His-tagged Proteins Is Strongly Dependent on the C2IIa Concentration*

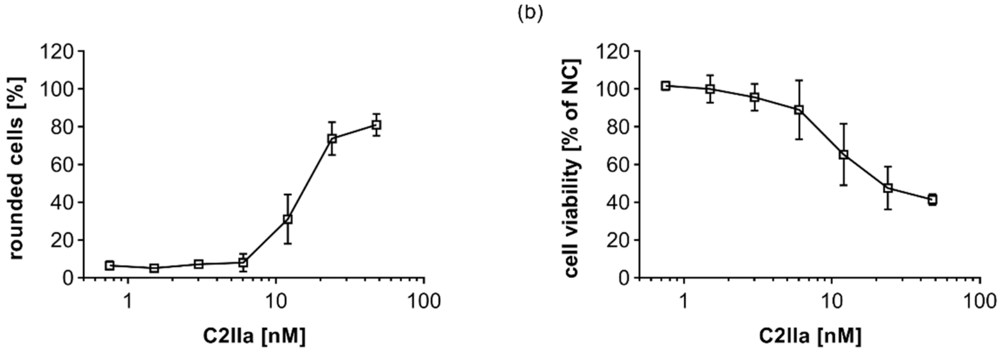

Figure S6. Cytosolic release of His_DTA is dependent on the C2IIa concentration. **(a)** HeLa cells were treated with a constant concentration of 250 nM His_DTA in combination with increasing concentrations of C2IIa (0.75 nM, 1.5 nM, 3 nM, 6 nM, 12 nM, 24 nM and 48 nM). Pictures of the cells were taken after 24 h and the amount of rounded cells was quantified as percentage of total cell number. Values are given as mean ± SD (*n* = 3) of triplicates from three individual experiments. **(b)** Relative viability (% of NC) of the cells from (a) measured via MTS assay. Values are given as mean ± SD (*n* = 3) of triplicates from three individual experiments.

1.5. *PA_{63}-Mediated Delivery of Lys_DTA Is Strongly Enhanced Compared to Transport of His_DTA*

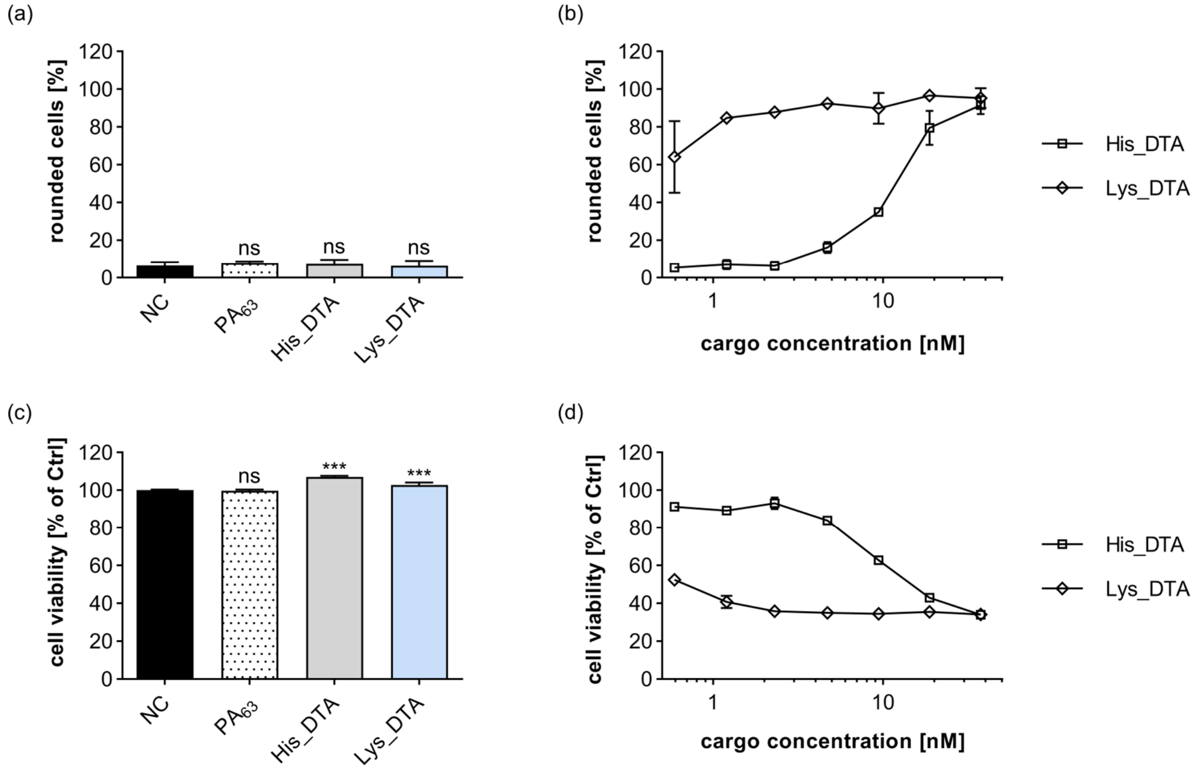

Figure S7. Lys_DTA is transported more efficiently into HeLa cells than His_DTA by PA_{63}. **(a)** HeLa cells were treated as indicated with 25 nM PA_{63}, 37.5 nM His_DTA or 37.5 nM Lys_DTA. Pictures of the cells were taken after 24 h and the amount of rounded cells was quantified as percentage of total cell number. Values are given as mean ± SD (*n* = 3) of triplicates from one experiment. **(b)** HeLa cells were treated with a constant concentration of 25 nM PA_{63} in combination with increasing concentrations of Lys_DTA and His_DTA (0.59 nM, 1.17 nM, 2.34 nM, 4.69 nM, 9.38 nM, 18.75 nM and 37.50 nM) within the same experiment as in (a). Pictures of the cells were taken after 24 h and the amount of rounded cells was quantified as percentage of total cell number. Values are given as mean ± SD (*n* = 3) of triplicates from one experiment. **(c)** Relative viability (% of NC) of the cells from (a) measured via MTS assay. Values are given as mean ± SD (*n* = 3) of triplicates from one experiment. **(d)** Relative viability (% of NC) of the cells from (b) measured via MTS assay. Values are given as mean ± SD (*n* = 3) of triplicates from one experiment. **(a, c)** Statistical analysis was performed compared to the NC by using non-parametric one-way ANOVA in combination with Dunnett's correction for multiple comparison as described under Section 5.12. in the Materials and Methods section (ns $p \geq 0.05$, *** $p < 0.001$).

1.6 *Direct Protein-Protein Interaction of Polycationic-Tagged DTA with C2IIa*

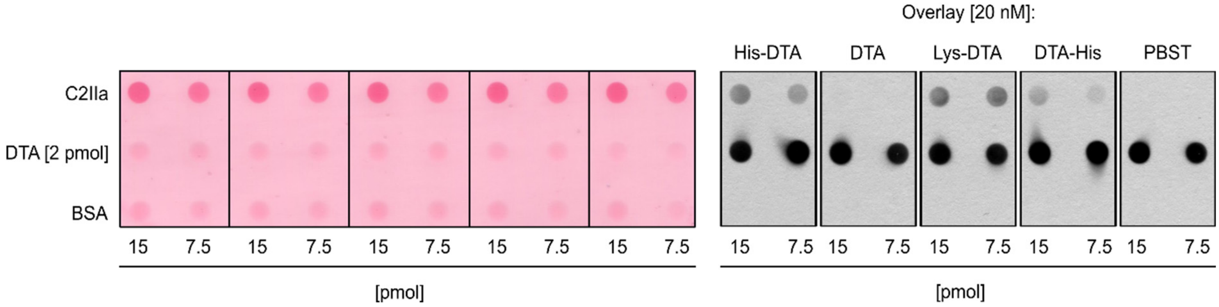

Figure S8. DTA variants only bind to C2IIa if a polycationic tag is present. DTA (2 pmol), C2IIa and BSA (15 pmol and 7.5 pmol, respectively) were vacuum-aspirated onto a nitrocellulose membrane as indicated using a dot blot device. In this setup, DTA served as positive control for antibody detection and BSA served as negative control to exclude unspecific binding. Spotting of the proteins was followed by membrane blocking with 5 % skim milk powder and an overlay with 20 nM of the indicated proteins in PBS-T or PBS-T only as control. Bound DTA variants were detected using a specific anti-diphtheria toxin (DTA) antibody. The second replicate of the experiment showed a similar result.

1.7. STED-Super Resolution Microscopy Unveils C2IIa-Mediated Uptake of Cargo into Early Endosomes

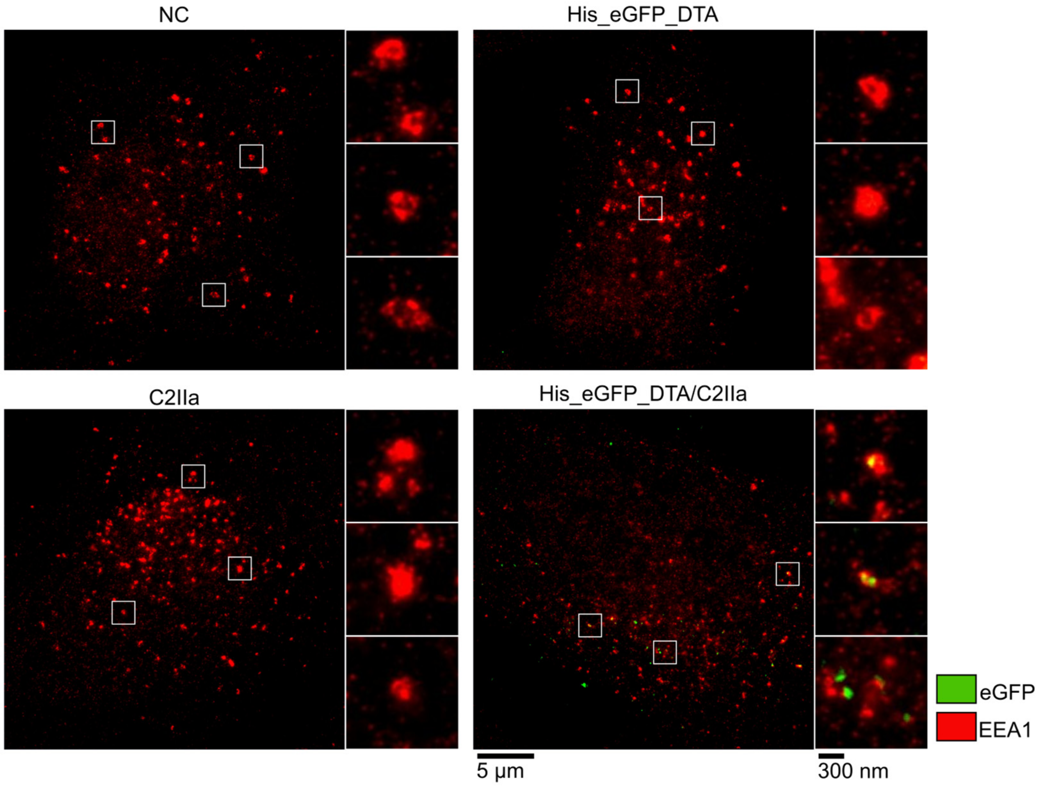

Figure S9. C2IIa mediates the uptake of His_eGFP_DTA into early endosomes. HeLa cells were treated with 250 nM His_eGFP_DTA alone, 25 nM C2IIa alone, the combination His_eGFP_DTA/C2IIa or left untreated (NC) for 30 min. STED super-resolution microscopy was performed and representative micrographs of the replicates are depicted with three magnified regions shown on the right side of each image (white squares indicate the respective areas in the main image). The signals for eGFP are shown in green, while EEA1-signals are depicted in red. Overlapping signals appear yellow. Scale bars correspond to 5 µm for the main images and 300 nm for the magnifications.

1.8. PA_{63} Delivers N-Terminally His-Tagged TcdB-GTD into the Cytosol of HeLa Cells

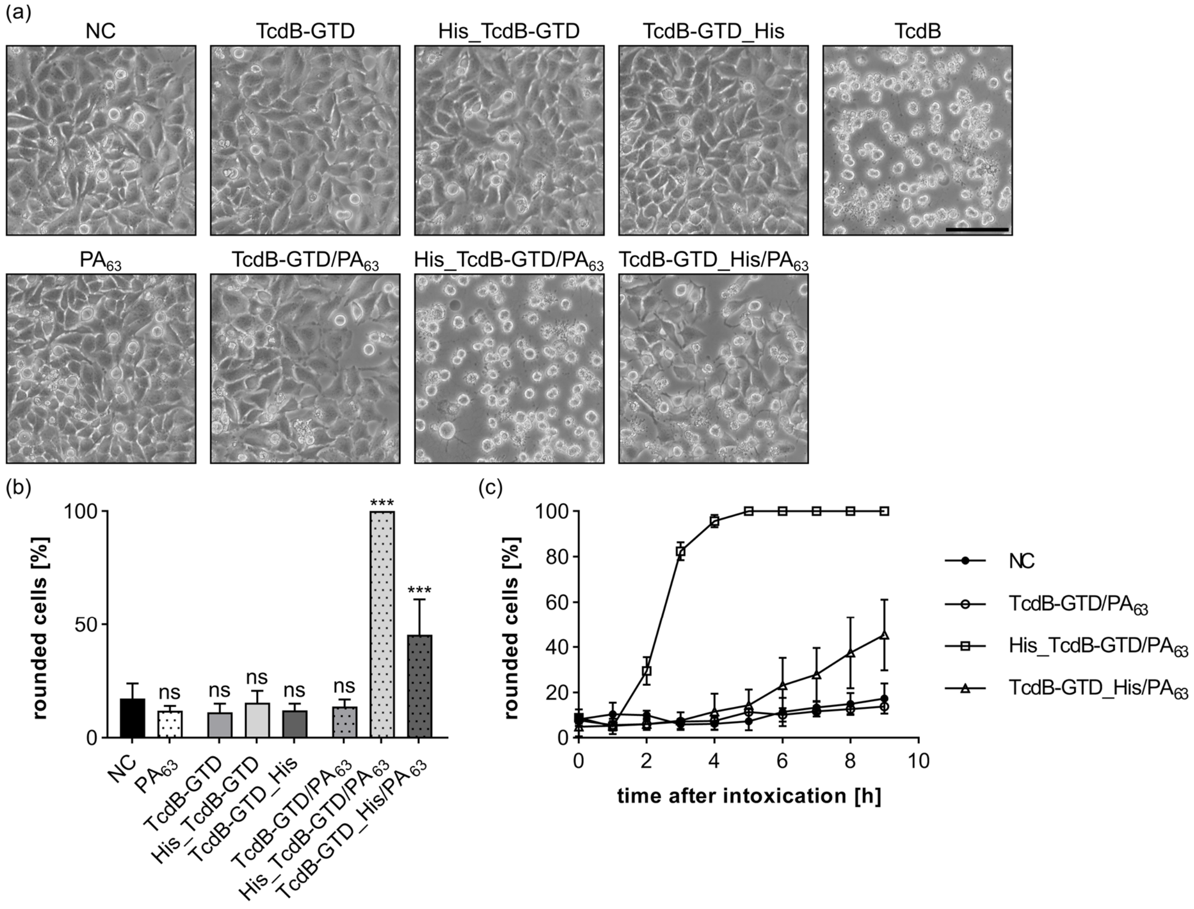

Figure S10. TcdB-GTD is efficiently transported into HeLa cells by PA_{63} in the presence of an N-terminal His-tag. **(a)** HeLa cells were treated as indicated with 10 nM PA_{63}, 100 nM TcdB-GTD cargo, 100 pM TcdB or without toxin (NC). Representative pictures after 10 h incubation time are depicted. Pictures were sampled from the same experiment as in **Figure 4a** and same assay controls are shown. Scale bar corresponds to 100 µm. **(b)** HeLa cells were treated as indicated with identical concentrations as in (a). The percentage of rounded cells at 9 h after intoxication is shown. Values are given as mean ± SD (n = 3) of a triplicate from one of three representative experiments. Statistical analysis was performed compared to the NC by using non-parametric one-way ANOVA in combination with Dunnett's correction for multiple comparison as described under Section 5.12. in the Materials and Methods section (ns $p \geq 0.05$, *** $p < 0.001$). The experiment was performed in parallel to the experiment in **Figure 4b** with the same controls. **(c)** Percentage of rounded cells from the same experiment as in (b) at different time points. Values are given as mean ± SD (n = 3) of a triplicate from one of three representative experiments.

1.9. Delivery of His_TcdB-GTD Is Dependent on Endosomal Acidification and C2IIa-Mediated Translocation

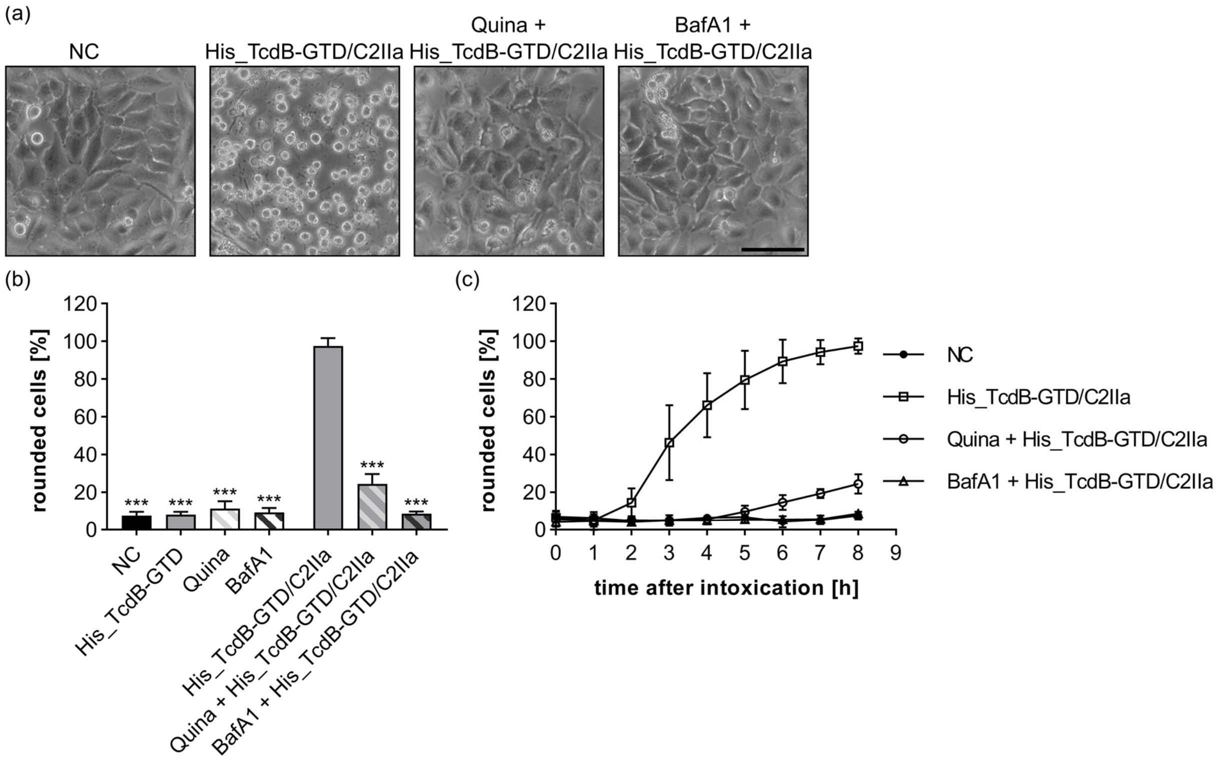

Figure S11. His_TcdB-GTD transport by C2IIa is inhibited by established inhibitors of C2IIa-mediated translocation. **(a)** HeLa cells were treated as indicated with 25 nM C2IIa, 200 nM His_TcdB-GTD, 5 µM Quinacrine (Quina), 100 nM Bafilomycin A1 (BafA1) or without toxin (NC). Representative pictures after 8 h incubation time are depicted. Scale bar corresponds to 100 µm. **(b)** HeLa cells were treated as indicated with identical concentrations as in (a). The percentage of rounded cells at 8 h after intoxication is shown. Additionally, His_TcdB-GTD, Quina and BafA1 controls are shown. Values are given as mean ± SD ($n = 3$) of a triplicate from one of three representative experiments. Statistical analysis was performed compared to the treatment with His_TcdB-GTD/C2IIa by using non-parametric one-way ANOVA in combination with Dunnett's correction for multiple comparison as described under Section 5.12. in the Materials and Methods section (*** $p < 0.001$). **(c)** Percentage of rounded cells from the same experiment as in (b) at different time points. Values are given as mean ± SD ($n = 3$) of a triplicate from one of three representative experiments.

1.10. C2IIa-Mediated Delivery of untagged TccC3hvr Is Reproducible on Vero Cells

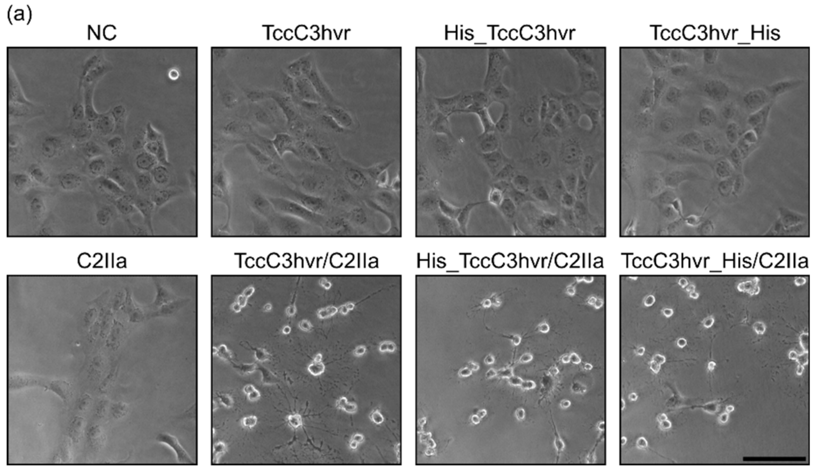

Figure S12. TccC3hvr constructs are transported into Vero cells by C2IIa. **(a)** Vero cells were treated as indicated with 10 nM C2IIa, 5 nM TccC3hvr cargo or without toxin (NC). Representative pictures after 4 h incubation time are depicted. Scale bar corresponds to 100 µm.

1.11. Delivery of His_TccC3hvr Is Dependent on Endosomal Acidification and C2IIa-Mediated Translocation

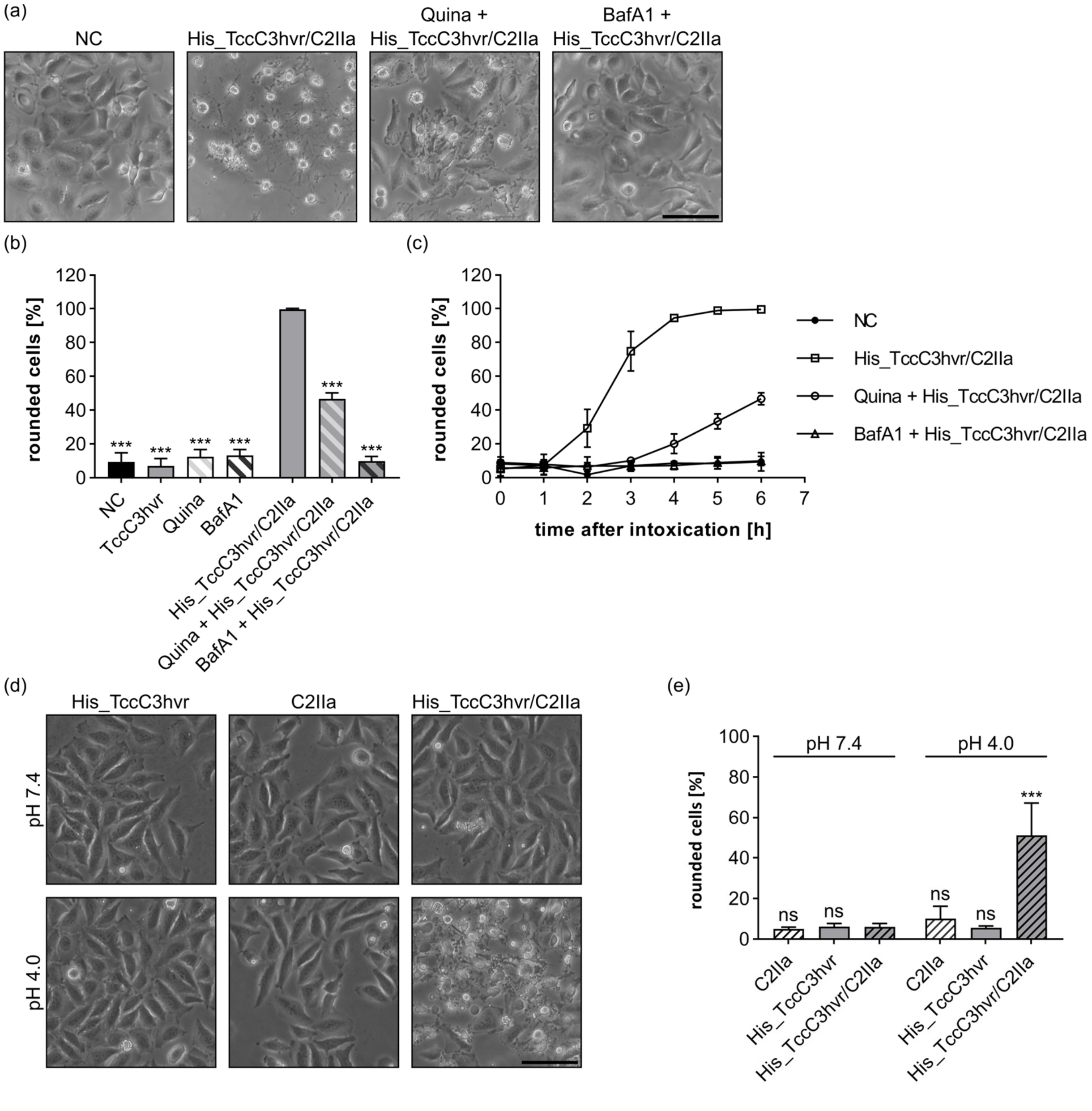

Figure S13. His_TccC3hvr transport by C2IIa is inhibited by established inhibitors of C2IIa-mediated translocation. **(a)** HeLa cells were treated as indicated with 10 nM C2IIa, 5 nM His_TccC3hvr, 5 µM Quinacrine (Quina), 100 nM Bafilomycin A1 (BafA1) or without toxin (NC). Representative pictures after 6 h incubation time are depicted. Scale bar corresponds to 100 µm. **(b)** HeLa cells were treated as indicated with identical concentrations as in (a). The percentage of rounded cells at 6 h after intoxication is shown. Additionally, His_TccC3hvr, Quina and BafA1 controls are shown. Values are given as mean ± SD ($n = 3$) of a triplicate from one of three representative experiments. Statistical analysis was performed compared to the treatment with His_TccC3hvr/C2IIa by using non-parametric one-way ANOVA in combination with Dunnett's correction for multiple comparison as described under Section 5.12. in the Materials and Methods section (*** $p < 0.001$). **(c)** Percentage of rounded cells from the same experiment as in (b) at different time points. Values are given as mean ± SD ($n = 3$) of a triplicate from one of three representative experiments. **(d)** Assay for translocation across the plasma membrane via acid pulse. HeLa cells were treated according to the assay protocol with 15 nM C2IIa and 50 nM His_TccC3hvr as indicated. Representative pictures taken 5 h after acidic pulse are depicted. Upper panel shows pictures of cells that were pulsed with serum free MEM pH 7.4 while the lower panel shows pictures of cells pulsed with serum free MEM pH 4.0. The other two biological replicates showed similar results. Scale bar corresponds to 100 µm. **(e)** Quantification of the assay for translocation from (d). Depicted is the percentage of rounded cells 5 h after acidic pulse. Values are given as mean ± SD ($n = 3$) of the biological replicates. Statistical analysis was performed compared to the treatment with His_TccC3hvr/C2IIa at pH 7.4 by using non-parametric one-way ANOVA in combination with Dunnett's correction for multiple comparison as described under Section 5.12. in the Materials and Methods section (ns $p \geq 0.05$, *** $p < 0.001$).

1.12. PA_{63} Delivers Proteins with Native Positively-Charged N-Terminus into the Cytosol of HeLa Cells

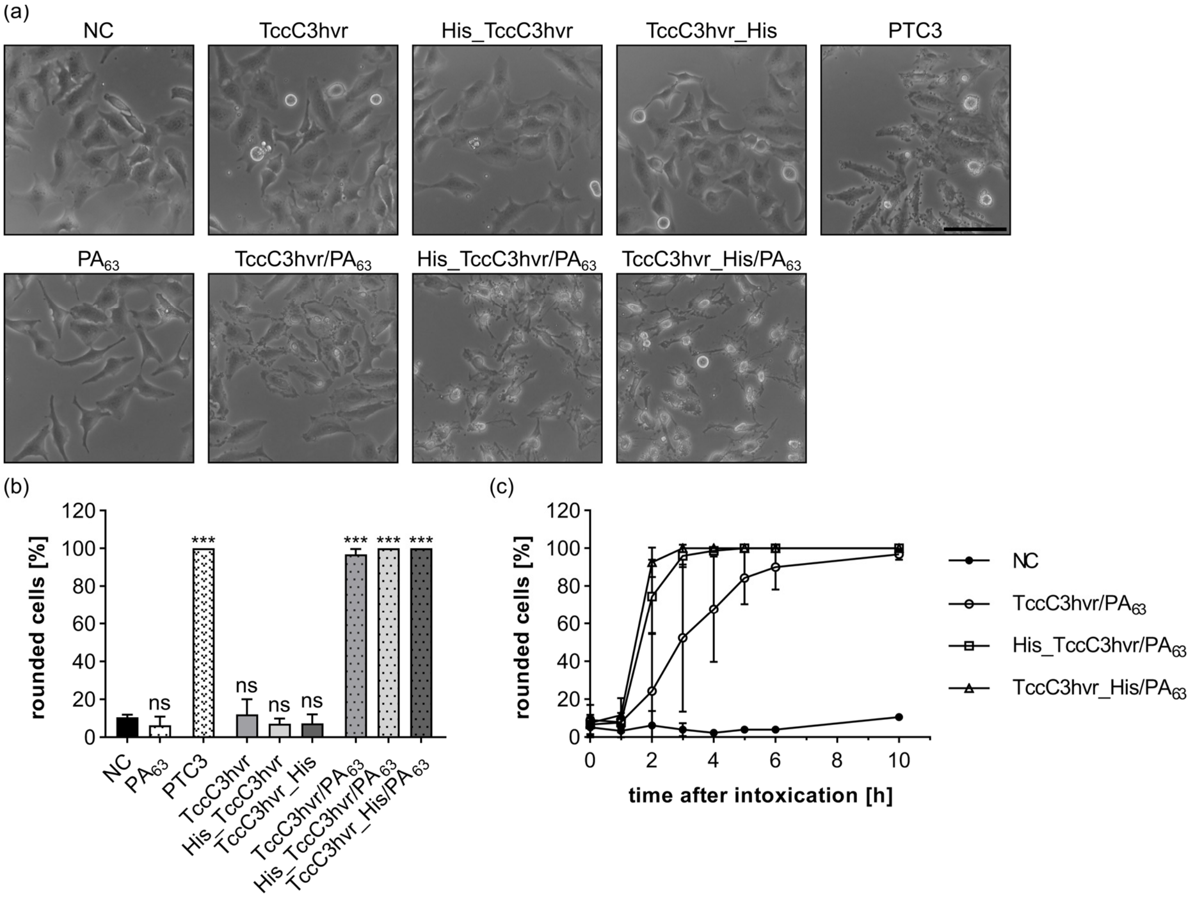

Figure S14. TccC3hvr constructs are efficiently transported into HeLa cells by PA_{63} independent of a His-tag. **(a)** HeLa cells were treated as indicated with 10 nM PA_{63}, 20 nM TccC3hvr cargo, PTC3 (1 µg/mL BC3 + 2 µg/mL TcdA1) or without toxin (NC). Representative pictures after 2 h incubation time are depicted. The experiment for the TccC3hvr constructs was performed in parallel to the experiments in **Figure 5**, **Figure 6**, and **Figure S15** and same assay controls are shown. Scale bar corresponds to 100 µm. **(b)** The percentage of rounded cells from the same experiment as in (a) at 10 h after intoxication is shown. Values are given as mean ± SD (n = 3) of a triplicate from one of three representative experiments. Statistical analysis was performed compared to the NC by using non-parametric one-way ANOVA in combination with Dunnett's correction for multiple comparison as described under Section 5.12. in the Materials and Methods section (ns $p \geq 0.05$, *** $p < 0.001$). **(c)** Percentage of rounded cells from (a) at different time points. Values are given as mean ± SD (n = 3) of a triplicate from one of three representative experiments.

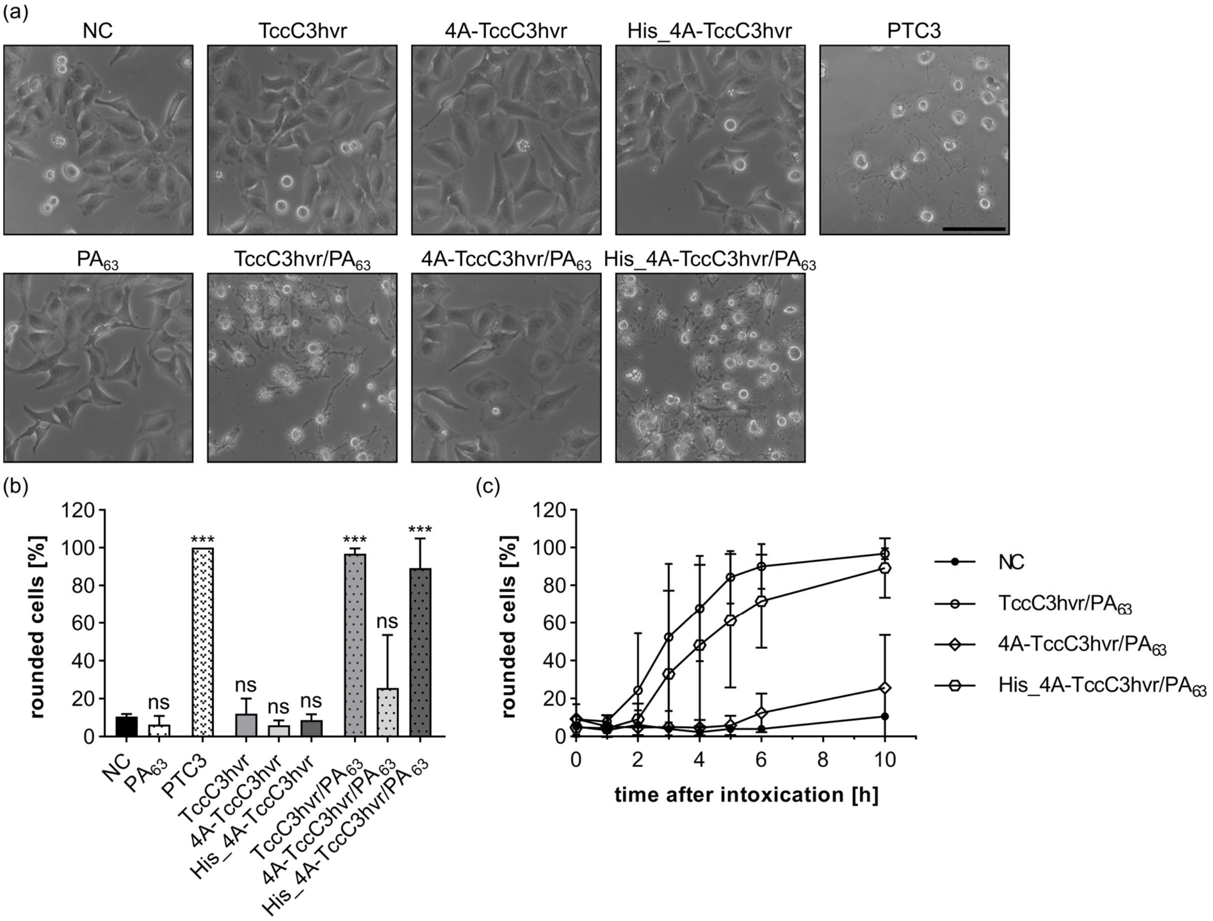

Figure S15. Transport of TccC3hvr by PA_{63} is dependent on the positive charge at the N-terminus of TccC3hvr. **(a)** HeLa cells were treated as indicated with 10 nM PA_{63}, 20 nM TccC3hvr cargo, PTC3 (1 µg/mL BC3 + 2 µg/mL TcdA1) or without toxin (NC). Representative pictures after 10 h incubation time are depicted. The experiment for the TccC3hvr constructs was performed in parallel to the experiments in **Figure 5**, **Figure 6**, and **Figure S14** and same assay controls are shown. Scale bar corresponds to 100 µm. **(b)** The percentage of rounded cells from the same experiment as in (a) at 10 h after intoxication is shown. Values are given as mean ± SD (n = 3) of a triplicate from one of three representative experiments. Statistical analysis was performed compared to the NC by using non-parametric one-way ANOVA in combination with Dunnett's correction for multiple comparison as described under Section 5.12. in the Materials and Methods section (ns $p \geq 0.05$, *** $p < 0.001$). **(c)** Percentage of rounded cells from (a) at different time points. Values are given as mean ± SD (n = 3) of a triplicate from one of three representative experiments.

2. Supplementary Materials and Methods

2.1. In vitro ADP-Ribosylation Assay

30 µg cell lysate dissolved in ADP-ribosylation buffer (20 mM Tris-HCl, 1 mM EDTA, 1 mM DTT, 5 mM $MgCl_2$, cOmplete™ (1:50, freshly added); pH 7.5) were incubated with either 25 pmol of TccC3hvr constucts or 5 pmol of DTA constructs together with 6-biotin-17-NAD^+ (10 µM) at 37 °C for 30 min. Laemmli buffer (0.3 M Tris-HCl, 10 % SDS, 37.5 % glycerol, 0.4 mM bromophenol blue, 100 mM DTT) was added to stop the ADP-ribosylation reaction and the samples were heat-denatured at 95 °C for 10 min. The amount of biotin-labelled toxin substrate was determined via SDS-PAGE and Western Blotting by using a streptavidin–peroxidase conjugate. Thus, a high enzymatic activity of the toxin results in a more intense signal detected in the Western Blot. For densitometric analysis of the protein bands ImageJ software (v1.51n, National Institute of Health, Bethesda, MD, USA) was used.

2.2. In vitro Glucosylation of Rac1 by TcdB-GTD

30 µg cell lysate was used as a Rac1 source. In addition to the cell lysate, 2 pmol of the TcdB-GTD variants were used in a total volume of 20 µl glucosylation buffer (50 mM HEPES, 100 mM KCl, 2 mM $MgCl_2$, 1 mM $MnCl_2$, 100 mg/l BSA, pH 7.5). Reactions were incubated at 37 °C for 90 min before they were stopped by addition of 5 µL Laemmlie Buffer and heat-denaturation (95 °C, 10 min). Samples were transferred to SDS-PAGE and Western Blotting. The non-glucosylated Rac1 signal of the Western blot was normalized to the Hsp90 loading control and given as a percentage of the negative control.

2.3. Dot Blot

The native proteins C2IIa, DTA and BSA were vacuum-aspired onto a nitrocellulose membrane with the indicated concentrations using a dot-blot manifold (Bio-Rad Laboratories, Inc., Hercules, CA, USA). After control of the transfer with Ponceau S the membrane was blocked for 1 h at RT with 5 % skimmed milk powder in PBS-T (137 mM NaCl, 2.7 mM KCl, 8 mM Na_2HPO_4, 1.8 mM KH_2PO_4, 0.1 % Tween20; pH 7.4). The overlay was performed with PBS-T or 20 nM of DTA, His-DTA, Lys-DTA or DTA-His in PBS-T, respectively, and incubated for 1 h at RT. After a washing step with PBS-T, the following antibody incubation, washing steps, and detection were performed according to the method section 5.10. SDS-PAGE and Western Blot.

2.4. Assay for Translocation at the Plasma Membrane via Acidic Pulse

4×10^4 cells per well were seeded two days prior to the assay in a 24-well plate. Cells were preincubated for 30 min with 100 nM Bafilomycin A1 (BafA1) in serum free MEM (also used for the other steps if not indicated otherwise) at 37 °C followed by a 10 min incubation on ice. The medium was exchanged to precooled toxin containing medium, which was again supplemented with 100 nM BafA1. Another incubation period of 30 min on ice allowed for toxin binding to the cell surface. Subsequently, the medium was removed and serum free MEM with either pH 7.4 or pH 4.0 was pipetted very carefully directly onto the cells. After a 10 min incubation time at 37 °C, the medium was again exchanged to serum containing MEM with pH 7.4 that was supplemented with 100 nM BafA1. Phase contrast microscopy pictures were taken at the indicated time points.
